# Evoked emotions in anorexia nervosa: neural and behavioural correlates of social-emotional processing

**DOI:** 10.1038/s41398-026-03819-8

**Published:** 2026-02-19

**Authors:** Jenni Leppanen, Olivia Bailey, Daniel Halls, Karina Allen, Kate Tchanturia, Steve Williams

**Affiliations:** 1 Department of Neuroimaging, Institute of Psychology, Psychiatry and Neuroscience, King’s College London, London, UK; 2 Research Unit of Clinical Medicine, Faculty of Medicine, University of Oulu, Oulu, Finland; 3 Centre for Clinical Brain Science, Division of Psychiatry, University of Edinburgh, Edinburgh, UK; 4 Eating Disorders Service, Maudsley Hospital, South London and Maudsley NHS Foundation Trust, London, UK; 5 Sir Peter Mansfield Imaging Centre, Mental Health and Clinical Neurosciences, School of Medicine, University of Nottingham, Nottingham, UK; 6 Department of Psychological Medicine, Institute of Psychiatry, Psychology and Neuroscience, King’s College London, London, UK; 7 Psychology Department, Illia State University, Tbilisi, Georgia

**Keywords:** Psychiatric disorders, Neuroscience

## Abstract

Previous work suggests people with anorexia nervosa (AN) display reduced facial expression of emotion. This may influence illness progression as blunted emotional reactions can negatively impact social relationships and increase isolation. The present study aimed to replicate and further build on previous findings by examining facial and brain responses to naturalistic, emotional films. In total, 141 women (71 AN/weight restored AN, 70 healthy comparison) completed two tasks in a fixed order: 1.) facial affect task and 2.) functional magnetic resonance imaging (fMRI) task. In both tasks, participants reacted to positive, neutral, and negative films, and rated their mood after each one. The effects of group and film category on facial expressions, brain responses, and mood ratings were examined. The AN group displayed reduced positive facial affect over time and lower self-reported mood in response to positive but not negative or neutral films. The fMRI task revealed no significant group differences in response to positive, neutral, or negative films. However, there was widespread activation of occipital, parietal, temporal, and frontal regions in response to the emotional films across groups. The behavioural findings replicate previously reported altered reactivity to positive films in AN. Additionally, task-related brain activation was observed in regions typically associated with the processing of naturalistic emotional stimuli, suggesting the task was valid. However, the lack of group differences during the fMRI task raises questions about whether the behavioural differences could be related to slower warming up to the task among those with AN.

## Introduction

Anorexia nervosa (AN) is a complex eating disorder (ED) characterised by restricted eating, body image disturbance, and inability to maintain a healthy body weight [1]. Theoretical models have suggested that difficulties in social-emotional processing, such as the reception and communication of emotions, play a key role in the progression of AN [2–4]. Experimental studies have documented that people with AN or lived experience of AN display reduced facial reactivity to naturalistic, emotional films [5]. Although this appears to be particularly pronounced in response to positive or amusing stimuli [5, 6], there is also evidence of reduced facial startle response to fear inducing films [7]. Reduced facial reactivity has also been reported to be associated with lower self-reported mood or subjective enjoyment [8, 9]. Such alterations in the reception of emotions and dampened emotional communication can have negative impact on social relationships and increase social isolation [4, 10].

Over the years, there has been increasing interest in exploring the neural underpinnings of emotional processing in AN [11, 12]. A recent meta-analysis of 19 functional magnetic resonance imaging (fMRI) studies compared whole brain responses to emotional stimuli between those with AN and healthy comparison (HC) participants [12]. The authors found increased activation in the cerebellum in people with AN compared to HC participants. Reduced blood oxygenation dependent (BOLD) response was identified in multiple regions including the striatum, parietal regions, and the frontal cortex, among those with AN. However, the studies included in the review used a variety of paradigms, some of which may be difficult to reconcile with real-world experiences (e.g., passive viewing of emotional images out of context) or may be linked to different underlying functions (e.g., guided emotion regulation) [11]. Therefore, although the findings were robust, it difficult to know to what extent they reflect the neural mechanisms underlying altered reception and dampened communication of emotions in AN.

Using similar naturalistic films inside the MRI environment as in the behavioural experiments could help explore the neural underpinnings of reduced facial reactivity and subjective enjoyment. Naturalistic film stimuli can also reflect the complexity of everyday social interactions, which cannot be captured in static pictures presented without context [13, 14]. Films can be used to differentiate between neural processes that underlie reception of emotional communication and those associated with self-reported emotional experiences [15]. Moreover, brain responses to naturalistic tasks generalise well to real-world experiences and have been used to predict social communication difficulties and friendships [16]. Still, to our knowledge, no studies to date have used such naturalistic paradigms to assess the neural underpinnings of social-emotional difficulties in AN.

The present study aimed to replicate previously reported reduced facial reactivity to emotional films and examine the neural underpinnings of such blunted responses. To achieve this, we developed two version of a task used to evoke emotional responses in and outside the MRI environment. We used previously evaluated films that were selected based on personal stories of significant positive and negative life events by people with lived experience of AN [17, 18]. Positive, neutral, and negative film clips were used to assess both behavioural and brain responses in women with AN and HC participants. Based on the previous work outlined above, we hypothesised that participants with AN would show reduced facial affect, specifically less positive expressivity, than the HC participants [5, 6]. Additionally, as we were evaluating a new, naturalistic fMRI task, we took a whole-brain approach to the fMRI analyses. Still, based on previous work, we hypothesised that we would observe group differences in the BOLD response in the occipital, parietal, and frontal regions [12].

## Materials and methods

### Participants

A total of 141 women (71 AN, 70 HC) took part in the study. All participants were MRI-compatible (not pregnant, no metal anywhere in or on the body that cannot be removed), right-handed women aged 18 – 26. All participants were screened using the Structured Clinical Interview for the DSM-5 – research version (SCID-5-RV) [19] and an MRI safety questionnaire to ensure eligibility. Participants were excluded if they reported any MRI incompatibility, history of or current substance abuse, a diagnosis of any neurological disorder, or history of head trauma.

Participants with AN were included if met DMS-5 criteria for AN on the SCID-5-RV screener, had or had had BMI under 18.5, had been diagnosed with AN by a psychiatrist or other health care professional and did not consider themselves to be recovered, regardless of current BMI status. All AN participants were recruited from the South London and Maudsley NHS Foundation Trust, South West London and St George’s Mental Health NHS Trust, the UK-based ED charity Beat (https://www.beateatingdisorders.org.uk/), and via adverts posted on social media (Facebook, Twitter/X). The HC participants were included if they did not report any current or past mental health problems on the SCID-5-RV. The HC participants were recruited through advertisements posted in and around King’s College London, through social media (Facebook, Twitter/X) and the local community.

All participants gave written informed consent before participating and were reimbursed £40 for their time. The study was approved by the London – South East Research Ethics Committee (Reference: 21/LO/0368), and all procedures were conducted according to the latest version of the Declaration of Helsinki (2013). This study was preregistered on the Open Science Framework database (10.17605/OSF.IO/2U8QG).

### Measures

#### Clinical and self-report assessments

Participants were asked to complete the following standardised self-report questionnaires to obtain information about the severity of current ED, anxiety, and depression symptoms: the Eating Disorder Examination Questionnaire (EDEQ) [20] (Cronbach’s alpha = 0.98) and the Hospital Anxiety and Depression Scale (HADS) [21] (Cronbach’s alpha = 0.94). Participants were also asked to complete a demographics questionnaire, and their height and weight were measured on the day to calculate body mass index (BMI; $\frac{{weight}({kg})}{{{height}(m)}^{2}})$.

#### Evoked emotions tasks

Two tasks, a Facial affect and an fMRI version, were developed to evoke positive and negative emotions using brief film clips (2.08 – 2.67 min). The Facial affect task was completed first and different film clips were used in the two tasks to minimise familiarity with the stimuli (Supplementary Table 1). After each task, participants were asked if they had seen any of the films previously and no participants reported familiarity with the stimuli.

The Facial affect task was used to assess facial expressions and self-reported mood. Participants facial expressions were video recorded while they watched two positive, two neutral, and two negative films presented in randomised order. After each film, participants were asked to rate their current mood on a scale from 0 to 100 using the Affective slider [22]. A fixation cross was presented for 5.2 s after the Affective slider, before the onset of the next film. The task was conducted using a laptop, and it was built using the Gorilla platform (https://gorilla.sc/).

The fMRI task was used to assess brain responses and self-reported mood. The task included two positive, two neutral, and two negative films presented in randomised order and after each film, participants rated their mood using the Affective slider. The duration of the fixation cross, presented after the Affective slider, was jittered (5.9 – 7.7 s). Noise-cancelling headphones were used to ensure participants could hear what was happening in the films.

### MRI data acquisition

The neuroimaging data were collected at the King’s College London Centre for Neuroimaging Sciences using a 3 Tesla GE Discovery MR750 scanner. A 12-channel head coil was used to accommodate the noise-cancelling headphones. The T1-weighted magnetisation-prepared rapid gradient echo (MPRAGE) anatomical images were acquired with the following parameters: 3.02 s echo time (TE), 7.31 s repetition time (RT), 11-degree flip angle, 256×256 matrix, 270 mm field of view, and 1.2 mm slice thickness. A T2*-weighted gradient echo, echo-planar imaging (EPI) sequence was used to collect functional BOLD signal with the following parameters: 0.03 s TE, 2 s TR, 75-degree flip angle, 64×64 matrix, 240 mm field of view, and 3 mm slice thickness. Full brain coverage was obtained with 41 slices, and 491 images were collected during the fMRI evoked emotions task.

### Data analysis

All code used in the data analysis is available at the study’s OSF repository (10.17605/OSF.IO/84MWQ).

#### Behavioural data preprocessing

Facial affect data were available from 64 HC participants and 70 AN participants due to video recording failure with four HC participants and one AN participant. Mood rating data were available from 68 HC participants and 71 AN participants due to issues with response recording with two HC participants.

Participants’ facial expressions were analysed using OpenFace [23, 24]. OpenFace, which uses Convolutional Expert Constrained Local Model to detect facial landmarks, can estimate the presence of 18 facial action units (AU) and provide a frame-wise intensity value for each AU on a scale from 0 (not present) to 5 (maximum intensity). We used AUs rather than basic emotions because many coding systems are based on posed emotions, but posed and spontaneous expressions have been shown to have different properties [25, 26]. We took the mean of two AUs typically associated with positive expressions, such as smiling, AU-6 (cheek raiser) and AU-12 (lip corner puller), and the mean of two AUs typically associated with negative expressions, such as frowning, AU-4 (brow lowerer) and AU-15 (lip corner depressor). The positive and negative AU time series were converted to a 0 – 1 scale using min-max scaling.

#### Statistical analysis of behavioural data

All statistical analyses of the behavioural data were conducted using R version 4.5.1 [27]. The significance level was set to p < 0.05 and the p-values were adjusted in the post-hoc comparisons using Bonferroni correction. Group differences in clinical and self-report measures were assessed with regression analyses.

The positive and negative AU time series were analysed with generalised additive mixed models (GAMMs using the *mgcv* package [28, 29]. We examined parametric effects of group, film category, and group by film category interaction as well as the smooth of time associated with each level of the group by film category interaction. The smooth functions were modelled using thing-plate splines [30] and the residuals were modelled using a first-order autoregressive (AR(1)) model. The full model with the interaction term was compared against a null model with only a time smooth and a no-interaction model. The model that offered the best fit to the observed data was selected using Akaike Information Criteria (AIC) and Χ^2^ -tests.

The Facial affect and fMRI task mood ratings were analysed with mixed effects regression models using the *glmmTMB* package [31, 32]. In both analyses, group, film category, and group by film category interactions were entered as predictors. The mood ratings were centred prior to analysis. The full models with the interaction term were compared against intercept-only null and no-interaction models using AIC and Χ^2^ -tests.

Finally, additional exploratory analyses were conducted within the AN group to examine the impact of clinical variables, BMI, EDEQ total scores, HADS anxiety scores, and HADS depression scores, on the outcomes. Separate GAMM analyses with clinical variable by films category interactions were conducted controlling for a smooth of time for each film category. Similarly, mixed effects analyses were conducted to examine the impact of clinical variable by film category interactions on the mood ratings while controlling for the impact of film category. The Bonferroni adjustment was used to account for the large number of exploratory tests and p < 0.003 was considered statistically significant.

#### FMRI data preprocessing

MRIQC was used to conduct quality assessment to identify acquisition errors [33]. One HC participant was excluded following the quality assessment due to significant shadow artefacts on the images. This resulted in a total number of 140 participants (71 AN, 69 HC) included in the fMRI data analyses. The preprocessing was conducted using fMRIprep [34, 35] with standard, minimal preprocessing steps for anatomical and functional images (Supplementary materials section 2).

#### First-level fMRI data analysis

The preprocessed time series data were modelled with *Nilearn* [36], adapted to include the inverse-logit (IL) hemodynamic response function (HRF) model [37] (Supplementary materials section 3). The residuals were modelled using an AR(1) noise model, and fMRIprep confounds and cosine high-pass filter included as nuisance covariates. The confounds included 24 head movement parameters and the CompCor parameters, which explained at least 50% of the variance due to noise arising from the cerebrospinal fluid (CSF). The high-pass filter cut-off was set at 294 s, which was obtained by multiplying the duration of the longest film clip by two to not remove low frequency signal in response to the stimuli [38]. The HRF response to each film was modelled separately, and t-contrasts were produced for each film using *Nilearn*. An additional mean response was calculated by taking the mean of each t-contrast.

We conducted additional exploratory analyses to examine connectivity during the fMRI task between regions of interest (ROIs) extracted from the Multi-Subject Dictionary Learning (MSDL) probabilistic brain atlas using *Nilearn*. The MSDL atlas consists of 39 brain regions, including the default mode network, insula, and areas of the temporal cortex, which make it suitable for movie-watching paradigms ([39]; Supplementary table 2). The time series from each ROI were averaged and denoised by using the above-mentioned confounds and high-pass filter. After denoising, functional connectivity between the ROIs while viewing each film was estimated by calculating lead-lag path signatures using *esig* [40, 41]. The signature method can be used to assess the relationship between two oscillatory paths and the lead-lag signature is used to analyse direction of the relationship [42–44].

#### Group-level fMRI data analysis

The group level analyses were conducted using Permutation Analysis of Linear Models (PALM) in FSL [45]. First, differences between the two groups were examined across the film categories by conducting a two-sample t-test using the mean t-contrast. Second, a mixed-effects model was used to investigate the effects of film category and film category by group interaction. Any significant findings from the mixed-effects models were explored using t-tests in PALM.

An exploratory mixed effects model was also used to examine the effects of film category and film category by group interaction on the lead-lag connectivity between the MSDL ROIs. Additional mixed effects exploratory analyses were conducted within the AN group to examine the impact of clinical variables, BMI, EDEQ total scores, HADS anxiety scores, and HADS depression scores, on the BOLD response. Finally, exploratory analyses examining the associations between subjective mood ratings and BOLD response and group by mood rating interaction are presented in the Supplementary materials section 5.

Exchangeability blocks were used in all within-subjects analyses to indicate which observations could be exchanged in the permutations to avoid inappropriate shuffling. To speed up the analyses, only the tails of the distributions were samples and 5000 permutations were used to obtain stable results. In the voxel-wise analyses, threshold-free cluster enhancement (TFCE) with family-wise error correction (FWER) was used to correct for multiple comparisons. In the ROI connectivity analysis, FWER was used to adjust the p-values for multiple comparisons. After FWER correction, p < 0.005 was used as the threshold for significance due to the number of tests.

## Results

### Sample characteristics

The sample demographic and clinical characteristics are presented in Table 1. The AN participants had lower BMI and reported higher scores on the EDEQ and HADS than the HC group. There were no significant differences between groups in age. Most AN participants had BMI < 18.5 at the time of participation (N = 47, 66%) and 43 (61%) reported having at least one comorbid diagnosis. The most common comorbid diagnoses were depression and anxiety disorders. Just over half (N = 36, 51%) of the AN participants reported taking psychiatric medication.

**Table 1:** Sample clinical and demographic characteristics.

Measure	Summary statistic	AN group (N = 71)	HC group (N = 70)	Z-statistic, p-value	ES [95% CI]
Age	Mean (SD)	21.24 (2.41)	21.53 (2.68)	z = −0.41, p = 0.584	−0.07 [−0.40, 0.26]
	Range	18 - 26	18 - 26		
BMI	Mean (SD)	17.65 (2.47)	21.67 (3.53)	z = 7. 32, p < 0.001	−1.32 [−1.68, −0.94]
	Range	11.06 - 25.36	15.81 - 33.42		
EDEQ Total	Mean (SD)	3.57 (1.39)	0.58 (0.67)	z = −13.73, p < 0.001	2.73 [2.26, 3.19]
	Range	0.15 - 5.61	0.00 - 3.96		
HADS Anxiety	Mean (SD)	12.07 (3.95)	5.04 (3.29)	z = −9.36, p < 0.001	1.92 [1.52, 2.33]
	Range	3.00 - 21.00	0.00 - 13.00		
HADS Depression	Mean (SD)	9.10 (4.51)	2.49 (2.60)	z = −9.12, p < 0.001	1.79 [1.40, 2.19]
	Range	1.00 - 18.00	0.00 - 11.00		
Comorbid diagnoses	N (%)	Depression: 24 (34%) Anxiety: 24 (34%) OCD: 9 (13%) Autism: 6 (8%) EUPD: 5 (7%) PTSD < 5 BD: < 5 BN: < 5 OSFED: < 5 OCPD: < 5 Trichotillomania: < 5	-	-	-
Psychiatric medication use (yes/no)	N (%)	Yes: 36 (51%) No: 33 (46%)	-	-	-

*BMI* body mass index, *EDEQ* eating disorder examination questionnaire, *HADS* hospital anxiety and depression scale, *AN* anorexia nervosa, *HC* healthy comparison, *OCD* obsessive compulsive disorder, *EUPD* emotionally unstable personality disorder, *BD* bipolar disorder, *BN* bulimia nervosa, *OSFED* other specified feeding or eating disorders, *OCPD* obsessive compulsive personality disorder, *SD* standard deviation, *ES* effect size (Hedge’s g), *CI* confidence interval.

Where diagnosis counts were less than 5, the actual counts are not reported to ensure individual participants cannot be identified.

### Facial affect evoked emotions task

#### Positive facial action units

The model comparisons indicated the full interaction GAMM offered best fit to the data (Supplementary table 4). There was a significant parametric effect of film category and group by film category interaction (Table 2). The post-hoc comparisons showed AN participants expressed more positive emotion during the positive films than neutral (t(110199) = 6.73, p < 0.001) or negative films (t(110199) = 11.48, p < 0.001). AN participants also expressed more positive emotion during the neutral films than negative films (t(110199) = 4.65, p < 0.001). Similarly, the HC participants expressed more positive emotion during the positive films than the neutral (t(110199) = 16.18, p < 0.001) or negative film (t(110199) = 17.65, p < 0.001). There was no significant difference in positive AU intensity between the negative and neutral films among the HC participants (t(110199) = 0.89, p > 0.999). There were no significant differences between the groups in positive AU intensity during positive (t(110199) = -0.71, p = 0.477), neutral (t(110199) = 0.53, p = 0.594), or negative films (t(110199) = 0.10, p = 0.919).

**Table 2:** Behavioural responses to the evoked emotions tasks.

Measure	Parametric terms	Estimate	SE	t statistic	p-value
Positive AUs	Group	−0.02	0.25	−0.08	0.936
	Film category (neutral)	0.08	0.03	3.12	0.002
	Film category (positive)	0.37	0.01	37.28	< 0.001
	Group x film category (neutral)	−0.02	0.04	−0.63	0.527
	Group x film category (positive)	0.28	0.01	19.62	< 0.001
	*Smooth terms*	*EDF*	*RDF*	*F statistic*	*p-value*
	Time: AN, positive films	11.63	13.09	6.34	< 0.001
	Time: HC, positive films	12.21	13.45	12.07	< 0.001
	Time: AN, neutral films	9.22	10.71	6.44	< 0.001
	Time: HC, neutral films	8.67	10.22	19.66	< 0.001
	Time: AN, negative films	8.81	10.56	3.79	< 0.001
	Time: HC, negative films	8.11	9.82	6.89	< 0.001
Negative AUs	*Parametric terms*	*Estimate*	*SE*	*t statistic*	*p-value*
	Group	−0.18	0.22	−0.83	0.405
	Film category (neutral)	−0.19	0.04	−4.42	< 0.001
	Film category (positive)	−0.18	0.01	−24.46	< 0.001
	Group x film category (neutral)	0.12	0.04	2.66	0.008
	Group x film category (positive)	−0.01	0.01	−0.48	0.628
	*Smooth terms*	*EDF*	*RDF*	*F statistic*	*p-value*
	Time: AN, positive films	12.40	13.55	9.41	< 0.001
	Time: HC, positive films	12.53	13.62	17.91	< 0.001
	Time: AN, neutral films	11.66	12.40	24.53	< 0.001
	Time: HC, neutral films	6.15	7.55	7.05	< 0.001
	Time: AN, negative films	12.29	13.45	14.18	< 0.001
	Time: HC, negative films	6.81	8.36	6.79	< 0.001
Mood ratings (Facial affect task)	*Fixed terms*	*Estimate*	*SE*	*z statistic*	*p-value*
	Group	0.06	0.09	0.70	0.481
	Film category (neutral)	1.04	0.07	14.48	< 0.001
	Film category (positive)	1.61	0.08	20.19	< 0.001
	Group x film category (neutral)	0.07	0.10	0.73	0.465
	Group x film category (positive)	0.33	0.11	3.06	0.002
Mood ratings (fMRI task)	*Fixed terms*	*Estimate*	*SE*	*z statistic*	*p-value*
	Group	0.15	0.05	2.92	0.004
	Film category (neutral)	1.03	0.05	19.43	< 0.001
	Film category (positive)	1.87	0.06	30.16	< 0.001

*AU* action unit, *SE* standard error, *EDF* effective degrees of freedom, *EDF* reference degrees of freedom.

**Fig. 1:**
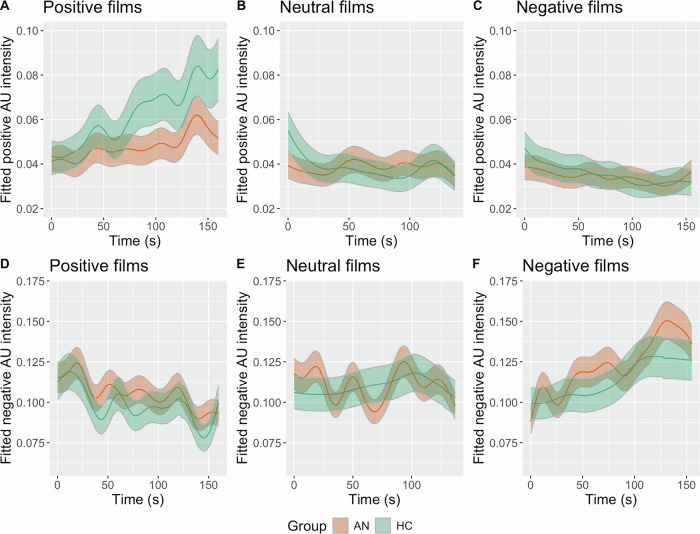
GAMM model smooth function of time for each level of the group by film category interactions. **A**. Smooth of positive AU intensity over time for the AN and HC groups during the positive films. **B**. Smooth of positive AU intensity over time for the AN and HC groups during the neutral films. **C**. Smooth of positive AU intensity over time for the AN and HC groups during the negative films. **D**. Smooth of negative AU intensity over time for the AN and HC groups during the positive films. **E**. Smooth of negative AU intensity over time for the AN and HC groups during the neutral films. **F**. Smooth of negative AU intensity over time for the AN and HC groups during the negative films. AU action unit, AN anorexia nervosa, HC healthy comparison.

The non-linear smooth effect of time was significantly different from zero for each level of the group by film category interaction (Table 2). Figure 1 shows that the confidence band of the HC and AN groups’ smooth functions does not overlap in the middle and towards the end of the positive films. This suggests that over time the HC participants expressed stronger positive reactions than the AN participants. The confidence interval bands overlapped across all time points during the neutral and negative films suggesting the groups did not significantly differ in the intensity of positive expressions over time.

#### Negative facial action units

The model comparisons favoured the full interaction GAMM over the other models (Supplementary table 5). There was a significant parametric effect of film category and group by film category interaction. The post-hoc comparisons showed that AN participants expressed more negative emotions during the negative films than neutral (t(110194) = 13.28, p < 0.001) or positive films (t(110194) = 8.73, p < 0.001). The AN group also expressed more negative emotion during the positive films than neutral films (t(110194) = 4.67, p < 0.001). The HC participants also expressed more negative emotion during the negative films than positive films (t(110194) = 5.72, p < 0.001) and during the neutral films than the positive films (t(110194) = 7.66, p < 0.001). There was no significant difference in the intensity of negative AUs during the negative and neutral films in the HC group (t(110194) = -2.33, p = 0.059). There were no significant differences between the groups in the intensity of negative AUs during the positive (t(110194) = 0.95, p = 0.344), neutral (t = -0.41, p = 0.683), or negative films (t(110194) = 1.30, p = 0.192).

The non-linear smooth functions were all significantly different from zero across all levels of the group by film category interaction (Table 2). Figure 1 shows that the smooths functions for the AN and HC groups overlap across all time points suggesting the groups did not significantly differ in the intensity of the negative AUs over time.

#### Mood ratings

The model comparison favoured the full interaction model over the other models (Supplementary table 6). There was a significant effect of film category and group by film category interaction. Both AN and HC participants rated their mood higher in response to the positive films than the neutral (AN: z = 7.50, p < 0.001; HC: z = 10.90, p < 0.001) or negative films (AN: z = 20.19, p < 0.001; HC: z = 23.86, p < 0.001). Both groups also rated their mood higher after the neutral films than the negative films (AN: z = 14.48, p < 0.001; HC: z = 15.47, p < 0.001). The HC participants rated their mood to be higher in response to the positive films than the AN group (z = 4.06, p < 0.001). There were no significant differences between the groups in mood ratings following the negative (z = 0.70, p = 0.481) or neutral films (z = 1.55, p = 0.121).

### FMRI evoked emotions task

#### Mood ratings

The model comparison favoured the no-interaction model over the other models (Supplementary table 7). The results showed a significant effect of group, such that the HC participants rated their mood higher than the AN participants across film categories (Table 2). There was also a significant effect of film category. Post-hoc comparisons showed that participants rated their mood higher following the positive film than the neutral (z = 14.03, p < 0.001) or negative films (z = 30.16, p < 0.001). Participants also reported more positive mood after the neutral films than the negative films (z = 19.43, p < 0.001).

Due to the different findings from the Facial affect and fMRI tasks, we conducted an additional exploratory analysis comparing the mood ratings from the two tasks. The results showed the expected significant effect of film category and group by film category interaction as well as a task by film category interaction (Supplementary table 8). The participants rated their mood higher after the positive films during the fMRI task than during the Facial affect task.

#### BOLD response

**Fig. 2:**
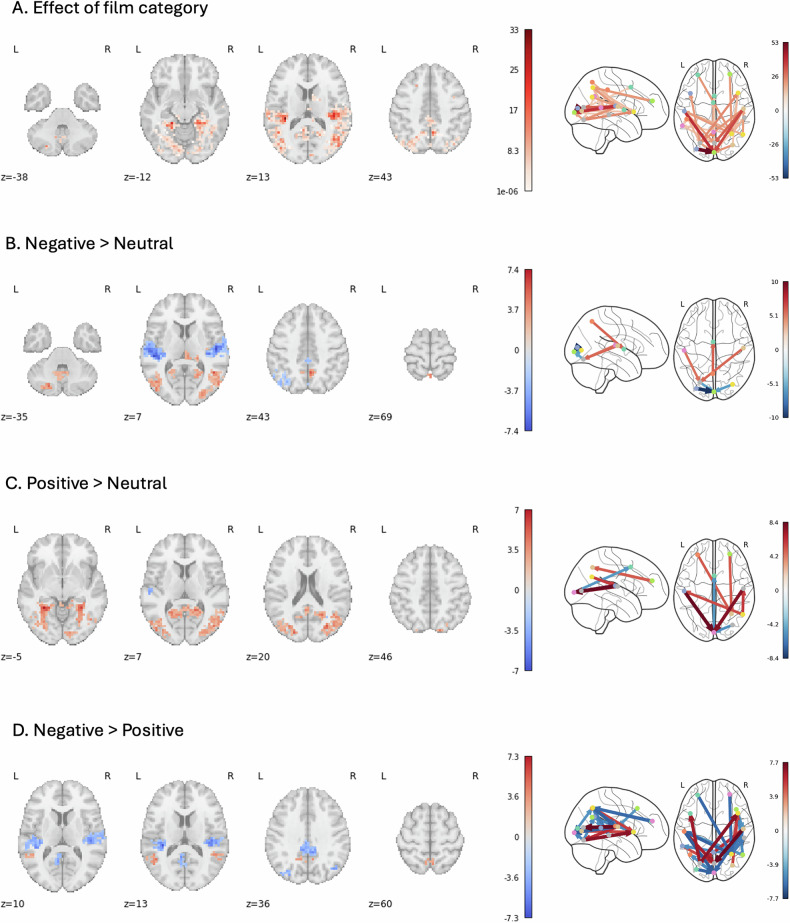
Effect of film category on BOLD response during the evoked emotions task. **A**. Axial view of F-statistic clusters showing the effect of film category, and sagittal and axial view of the film category connectome. **B**. Axial view of t-statistic clusters showing Negative > Neutral film category post-hoc comparison, and sagittal and axial view of the Negative > Neutral film category connectome. Positive t-statistics indicate greater BOLD response to negative films than neutral films. Negative t-statistics indicate greater BOLD response to neutral films than negative films. Positive connections indicate stronger connectivity during the negative films than neutral films. Negative connections indicate stronger connectivity during the neutral films than negative films. **C**. Axial view of t-statistic clusters showing Positive > Neutral film category post-hoc comparison, and sagittal and axial view of the Positive > Neutral film category connectome. Positive t-statistics indicate greater BOLD response to positive films than neutral films. Negative t-statistics indicate greater BOLD response to neutral films than positive films. Positive connections indicate stronger connectivity during the positive films than neutral films. Negative connections indicate stronger connectivity during the neutral films than positive films. **D**. Axial view of t-statistic clusters showing Negative > Positive film category post-hoc comparison, and sagittal and axial view of the Negative > Positive film category connectome. Positive t-statistics indicate greater BOLD response to negative films than positive films. Negative t-statistics indicate greater BOLD response to positive films than negative films. Positive connections indicate stronger connectivity during the negative films than positive films. Negative connections indicate stronger connectivity during the positive films than negative films. All results are shown at p < 0.005 after TCFE or cross-contrast (lead-lag signatures) FWER-correction with p < 0.005.

There was a significant effect of film category on the BOLD response during the fMRI evoked emotions task in several regions across the occipital, temporal and frontal cortices (Table 3, Fig. 2; Supplementary figure 2). There were no significant group differences or group by film category interaction.

**Table 3:** Mixed model results for fMRI evoked emotions task.

	Cluster	Peak coordinates			Cluster size (mm^3^)	Test statistics		Cluster mean			Regions (AAL)
		X	Y	Z		Peak	Cluster mean	AN	HC	ES [95% CI]	
Effect of film category	1	−47.8	−42.5	0.7	174441	F = 8.41, p < 0.001	F = 5.43, p < 0.001	0.1 (0.40)	0.08 (0.45)	0.03 [−0.30, 0.36]	L Middle Occipital cortex
											R Superior temporal cortex
		38.5	−12.5	4							L Middle temporal cortex
		38.5	−72.5	43.6							R Middle Temporal Cortex
		38.5	−42.5	−12.5							L Precuneus
	2	−25.3	−76.3	−38.9	510	F = 14.50, p < 0.001	F = 8.24, p = 0.001	0.52 (1.03)	0.4 (1.10)	0.11 [−0.23, 0.44]	L Cerebellum Crus II
											L Cerebellum Crus I
	3	8.5	−61.3	−42.2	696	F = 14.65, p < 0.001	F = 6.28, p = 0.003	0.16 (0.99)	−0.02(0.95)	0.18 [−0.15, 0.51]	R Vermis VIII
											R Cerebellum
		−2.75	−68.8	−42.2							R Vermis VIIII
	4	−6.5	51.3	27.1	1902	F = 12.80, p < 0.001	F = 7.05, p < 0.001	0.03 (0.65)	−0.06 (0.73)	0.13 [−0.21, 0.46]	L Superior frontal cortex
		−6.5	58.8	30.4							R Superior frontal cortex
		1	58.8	27.1							
		8.5	66.3	27.1							
	5	42.3	2.5	53.5	649	F = 10.22, p < 0.001	F = 7.31, p = 0.001	−0.06 (1.21)	−0.16 (1.15)	0.08 [−0.25, 0.41]	R Precentral cortex
											R Middle frontal cortex
	6	−6.5	−61.3	−42.2	417	F = 9.37, p < 0.001	F = 6.66, p = 0.002	0.2 (0.78)	0.05 (0.84)	0.18 [−0.16, 0.51]	L Cerebellum
	7	4.8	−91.3	13.9	510	F = 9.47, p < 0.001	F = 6.47, p = 0.002	−0.37 (1.25)	−0.36 (1.27)	−0.01 [−0.34, 0.33]	L Cuneus
											L Calcarine cortex
											R Calcarine cortex
											R Cuneus
	8	−29	10	56.8	556	F = 11.33, p < 0.001	F = 8.07, p = 0.001	−0.04 (1.12)	−0.11 (1.18)	0.06 [−0.27, 0.39]	L Middle frontal cortex
		−21.5	17.5	46.9							L Superior frontal cortex
	9	−21.5	−35	7.3	417	F = 10.0, p < 0.001	F = 5.28, p = 0.003	−0.25 (0.73)	−0.32 (0.86)	0.08 [−0.25, 0.42]	L Thalamus
		−14	−31.3	10.6							L Hippocampus
	10	−17.8	−50	−45.5	417	F = 9.03, p < 0.001	F = 7.32, p = 0.002	−0.1 (0.63)	−0.11 (0.74)	0.03 [−0.31, 0.36]	L Cerebellum
		−10.3	−50	−48.8							
Post-hoc comparisons											
Negative > Neutral	1	−47.8	−23.8	7.3	11369	t = −6.24, p < 0.001	t = −3.91, p =	−0.51 (0.61)	−0.45 (0.78)	−0.08 [−0.41, 0.26]	L Superior temporal cortex
		−51.5	−8.8	4							L Heschl’s gyrus
		−59	−1.6	4							
		−51.5	−38.8	13.9							
	2	46	−80	−2.6	22460	t = 4.58, p < 0.001	t = 2.96, p < 0.001	0.47 (0.92)	0.44. (0.94)	0.04 [−0.30, 0.37]	R Middle temporal cortex
											R Middle occipital cortex
		42.3	−65	−2.6							R Superior temporal cortex
		42.3	−68.8	17.2							R Supramarginal gyrus
		46	−61.3	−2.6							R Angular gyrus
	3	49.8	−16.3	7.3	12761	t = −7.38, p < 0.001	t = −3.48, p < 0.001	−0.43 (0.57)	−0.35 (0.67)	−0.12 [−0.45, 0.21]	R Superior temporal cortex
		42.3	−20	0.7							R Rolandic operculum
		57.3	−27.5	4							R Heschl’s gyrus
		64.8	−16.3	−2.6							R Insula
	4	−40.3	−68.8	50.2	5847	t = −3.91, p < 0.001	t = −2.61, p = 0.002	−0.50 (0.86)	−0.44 (1.03)	−0.06 [−0.39, 0.28]	L Inferior parietal cortex
		−47.8	−65	50.2							L Angular gyrus
		−55.3	−61.3	46.9							L Superior parietal cortex
		−47.8	−72.5	40.3							L Middle occipital cortex
	5	−2.8	55	27.1	4083	t = 3.61, p < 0.001	t = 3.16, p = 0.001	0.63 (1.34)	0.63 (1.28)	−0.002 [−0.33, 0.33]	L Superior frontal cortex
		1	62.5	30.4							R Superior frontal cortex
		−6.5	51.3	33.7							
		1	66.3	20.5							
	6	−2.8	−53.8	56.8	6960	t = 3.82, p < 0.001	t = 2.97, p < 0.001	0.72 (1.15)	0.47 (1.26)	0.20 [−0.13, 0.54]	R Precuneus
		1	−57.5	46.9							L Precuneus
		12.3	−46.3	56.8							
		−14	−50	60.1							
	7	−10.3	−42.5	33.7	3155	t = −4.07, p < 0.001	t = −2.96, p = 0.001	−0.41 (1.03)	−0.54 (1.36)	0.11 [−0.22, 0.44]	L Middle cingulate
		−2.8	−38.8	33.7							R Middle cingulate
		−2.8	−38.8	23.8							L Posterior cingulate
		−2.8	−31.3	40.3							
	8	−47.8	−72.5	10.6	28354	t = 3.27, p < 0.001	t = 2.72, p < 0.001	0.45 (0.68)	0.38 (0.77)	0.09 [−0.24, 0.42]	L Middle occipital cortex
		−59	−53.8	17.2							L Middle temporal cortex
		−55.3	−61.3	10.6							L Lingual gyrus
		−51.5	−50	17.2							L Fusiform gyrus
	9	19.8	−57.5	10.6	1531	t = 4.57, p < 0.001	t = 3.16, p = 0.001	0.49 (1.07)	0.38 (1.08)	0.11 [−0.24, 0.42]	R Calcarine cortex
		27.3	−61.3	20.5							R Precuneus
		16	−50	4							R Cuneus
	10	19.8	−83.8	−15.8	3758	t = 4.14, p < 0.001	t = 1.86, p = 0.001	0.36 (1.14)	0.25 (1.31)	0.09 [−0.25, 0.42]	R Lingual gyrus
											R Fusiform gyrus
		19.8	−87.5	−5.9							R Calcarine cortex
		23.5	−80	−−9.2							R Cerebellum
		8.5	−91.3	−5.9							R Inferior occipital cortex
	11	19.8	−38.8	−9.2	2877	t = 4.22, p < 0.001	t = 2.80, p = 0.001	0.31 (0.85)	0.37 (0.82)	−0.8 [−0.41, 0.25]	R Fusiform gyrus
		31	−46.3	−5.9							R Parahippocampus
		19.8	−50	−12.5							R Lingual gyrus
	12	−17.8	−50	−45.5	6543	t = 4.14, p < 0.001	t = 2.61, p = 0.002	0.32 (0.62)	0.24 (0.72)	0.12 [−0.21, 0.45]	L Inferior paraietal cortex
		−10.3	−50	−48.8							L Angular gyrus
		−6.5	−61.3	−42.2							L Superior parietal cortex
		−10.3	−53.8	−38.9							L Middle occipital cortex
	13	31	−57.5	−19.1	3294	t = 4.07, p < 0.001	t = 2.70, p = 0.002	0.32 (0.78)	0.31 (0.74)	0.01 [−0.33, 0.34]	R Fusiform gyrus
		34.8	−50	−15.8							R Inferior temporal cortex
		42.3	−53.8	−12.5							R Cerebellum
		38.5	−65	−15.8							R Inferior occipital cortex
	14	4.8	−27.5	7.3	2459	t = 3.87, p < 0.001	t = 2.84, p = 0.002	0.32 (0.77)	0.28 (0.85)	0.05 [−0.28, 0.38]	R Thalamus
		4.8	−38.8	0.7							
		19.8	−31.3	7.3							
		4.8	−20	10.6							
	15	−6.5	−72.5	37	881	t = −3.94, p = 0.001	t = −2.73, p = 0.003	−0.38 (1.38)	−0.61 (1.49)	0.16 [−0.18, 0.49]	L Precuneus
		−14	−65	37							L Cuneus
		−6.5	−65	33.7							
		−10.3	−68.8	27.1							
	16	31	−80	37	464	t = 4.16, p = 0.001	t = 2.90, p = 0.003	0.76 (1.61)	0.37 (1.70)	0.24 [−0.09, 0.57]	R Middle occipital cortex
											R Superior occipital cortex
	17	−36.5	−76.3	30.4	278	t = −3.42, p = 0.002	t = −2.68, p = 0.003	−0.56 (1.70)	−0.43 (1.44)	−0.08 [−0.41, 0.26]	L Middle occipital cortex
											L Angular gyrus
	18	−6.5	−80	50.2	464	t = −3.46, p = 0.002	t = −2.80, p = 0.003	−0.59 (1.67)	−0.46 (1.55)	−0.08 [−0.41, 0.25]	L Precuneus
	19	8.5	2.5	17.2	278	t = 3.46, p = 0.002	t = 3.12, p = 0.003	0.44 (1.09)	0.35 91.36)	0.08 [−0.25, 0.41]	R Caudate
Positive > Neutral	1	−40.3	−80	13.9	73832	t = 3.28, p < 0.001	t = 2.81, p < 0.001	0.47 (0.66)	0.37 (0.64)	0.16 [−0.18, 0.49]	L Middle occipital cortex
											R Middle occipital cortex
		−51.5	−68.8	−2.6							R Middle temporal cortex
		−36.5	−83.8	4							R Fusiform gyrus
		−40.3	−76.3	−5.9							L Fusiform gyrus
	2	−66.5	−16.3	10.56	649	t = −5.02, p < 0.001	t = −3.75, p = 0.002	−0.55 (0.96)	−0.37 (1.24)	−0.16 [−0.49, 0.17]	L Superior temporal cortex
		−59	−20	7.3							L Heschl’s gyrus
		−51.5	−20	4							
Negative > Positive	1	−36.5	−31.3	13.9	6404	t = −7.28, p < 0.002	t = −3.47, p < 0.001	−0.43 (0.84)	−0.42 (0.88)	−0.01 [−0.34, 0.33]	L Superior temporal cortex
		−44	−31.3	7.3							L Heschl’s gyrus
		−36.5	−27.5	7.3							L Rolandic operculum
		−47.8	−20	7.3							L Middle temporal cortex
	2	42.3	−23.8	13.9	6496	t = −5.71, p < 0.001	t = −3.34, p < 0.001	−0.39 (0.79)	−0.37 (0.79)	−0.02 [−0.35, 0.31]	R Superior temporal cortex
		49.8	−12.5	7.3							R Heschl’s gyrus
		64.8	−20	10.6							R Rolandic operculum
		57.3	−27.5	7.3							R Insula
	3	−6.5	−38.8	33.7	9745	t = −4.94, p < 0.001	t = −3.16, p = 0.002	−0.51 (0.91)	−0.50 (1.08)	−0.01 [−0.34, 0.33]	R Middle cingulate
											L Middle Cingulate
		1	−42.5	33.7							L Precuneus
		8.5	−38.8	40.3							L Posterior cingulate
		−6.5	−53.8	17.2							L Calcarine cortex
	4	−47.75	−53.8	13.9	3619	t = 4.72, p < 0.001	t = 3.17, p = 0.002	0.55 (1.24)	0.42 (1.16)	0.11 [−0.23, 0.44]	L Middle temporal cortex
		−51.5	−57.5	23.8							L Angular gyrus
		−59	−53.8	17.2							
		−51.5	−61.3	13.9							
	5	−2.8	−57.5	50.2	4037	t = 4.37, p < 0.001	t = 3.05, p < 0.001	0.67 (1.33)	0.60 (1.49)	0.05 [−0.28, 0.38]	L Precuneus
		−10.3	−53.8	43.6							R Precuneus
		4.8	−57.5	56.8							
		4.8	−53.8	43.6							
	6	−32.8	−68.8	46.9	3851	t = −5.00, p < 0.001	t = −3.00, p < 0.001	−0.60 (0.80)	−0.55 (1.21)	−0.04 [−0.37, 0.29]	L Middle occipital cortex
		−21.5	−61.3	43.6							L Inferior parietal cortex
		−29	−80	37							L Superior parietal cortex
		−44	−76.3	37							L Angular gyrus
	7	64.8	−42.5	23.8	3062	t = 4.70, p < 0.001	t = 2.89, p = 0.002	0.47 (1.12)	0.42 (1.06)	0.04 [−0.29, 0.37]	R Superior temporal cortex
		46	−46.3	27.1							R Supramarginal gyrus
		53.5	−50	23.8							R Angular gyrus
		46	−46.3	13.9							R Middle temporal cortex
	8	42.3	−68.8	37	464	t = −4.80, p < 0.001	t = −3.97, p = 0.001	−0.55 (1.57)	−0.91 (1.78)	0.21 [−0.12, 0.55]	R Angular gyrus

Cluster mean in the effect of film category show cluster mean intensity across film categories for each group. Cluster means in post-hoc comparisons show difference in cluster mean BOLD intensity between the film categories in the contrast for each group. The effect size shows the size of the group difference.

*AAL* automated anatomical labelling atlas, *ES* effect size, *CI* confidence interval.

The post-hoc comparisons revealed 12 clusters across multiple regions, including the occipital, temporal, and superior frontal cortices, which showed stronger BOLD response to the negative than the neutral films (Table 3, Fig. 2; Supplementary figure 3). There were also seven clusters in the right temporal cortex, right insula, and left cingulate cortex, which showed stronger BOLD response to the neutral than the negative films. One cluster, which covered occipital and temporal regions, showed a stronger BOLD response to the positive than the neutral films. One cluster in the right superior temporal cortex showed stronger response to the neutral than the positive films. Three clusters in the temporal and posterior regions were more active during the negative than positive films. Five other clusters, which included the right insula, cingulate cortex, and parietal regions, showed stronger BOLD response during the positive than the negative films.

#### Functional connectivity

The exploratory connectivity results echoed those outlined above showing a significant effect of film category and no significant effect of group or group by film category interaction (Fig. 2, Supplementary table 9). During the positive and negative films, the activation flowed from the temporal to posterior visual regions. The activation also flowed from the visual to parietal regions during the positive films and to the temporal cortex and insula during the negative films. Additionally, during the positive films, connectivity from the anterior insula and the anterior cingulate to the inferior parietal cortex was observed. No clusters were identified in these anterior regions when comparing peak HRFs.

### Impact of clinical variables on evoked emotions task responses in the AN group

#### Facial action units

There were no significant parametric effects of any clinical variables on the intensity of the positive or negative AUs within the AN group (Table 4).

**Table 4:** Impact of clinical variables on behavioural responses to the evoked emotions tasks.

*Measure*	Clinical variable	*Parametric terms*	*Estimate*	*SE*	*t statistic*	*p-value*
Positive AUs	BMI	BMI x film category (positive)	−0.003	0.07	−0.05	0.962
		BMI x film category (neutral)	−0.02	0.07	−0.26	0.794
		BMI x film category (negative)	−0.03	0.07	−0.36	0.720
		*Smooth terms*	*EDF*	*RDF*	*F statistic*	*p-value*
		Time: positive films	11.67	13.12	7.36	< 0.001
		Time: neutral films	9.28	10.84	6.87	< 0.001
		Time: negative films	9.08	10.84	3.87	< 0.001
	EDEQ total	*Parametric terms*	*Estimate*	*SE*	*t statistic*	*p-value*
		EDEQ x film category (positive)	0.16	0.12	1.30	0.194
		EDEQ x film category (neutral)	0.09	0.12	0.75	0.453
		EDEQ x film category (negative)	0.07	0.12	0.54	0.588
		*Smooth terms*	*EDF*	*RDF*	*F statistic*	*p-value*
		Time: positive films	11.56	13.04	6.38	< 0.001
		Time: neutral films	9.07	10.75	6.06	< 0.001
		Time: negative films	9.33	11.09	4.97	< 0.001
	HADS anxiety	*Parametric terms*	*Estimate*	*SE*	*t statistic*	*p-value*
		HADS anxiety x film category (positive)	0.01	0.04	0.33	0.739
		HADS anxiety x film category (neutral)	−0.003	0.04	−0.07	0.944
		HADS anxiety x film category (negative)	−0.01	0.04	−0.27	0.789
		*Smooth terms*	*EDF*	*RDF*	*F statistic*	*p-value*
		Time: positive films	11.36	12.90	6.19	< 0.001
		Time: neutral films	9.26	10.93	5.82	< 0.001
		Time: negative films	9.49	11.24	5.51	< 0.001
	HADS depression	*Parametric terms*	*Estimate*	*SE*	*t statistic*	*p-value*
		HADS depression x film category (positive)	0.02	0.04	0.52	0.606
		HADS depression x film category (neutral)	−0.005	0.04	−0.12	0.906
		HADS depression x film category (negative)	−0.01	0.04	−0.17	0.865
		*Smooth terms*	*EDF*	*RDF*	*F statistic*	*p-value*
		Time: positive films	11.40	12.93	5.99	< 0.001
		Time: neutral films	9.32	10.99	7.11	< 0.001
		Time: negative films	10.18	11.88	7.20	< 0.001
Negative AUs	BMI	*Parametric terms*	*Estimate*	*SE*	*t statistic*	*p-value*
		BMI x film category (positive)	0.0002	0.05	0.005	0.996
		BMI x film category (neutral)	−0.006	0.05	−0.11	0.915
		BMI x film category (negative)	0.01	0.05	0.19	0.847
		*Smooth terms*	*EDF*	*RDF*	*F statistic*	*p-value*
		Time: positive films	12.17	13.43	7.95	< 0.001
		Time: neutral films	12.19	13.00	22.98	< 0.001
		Time: negative films	11.98	13.27	12.61	< 0.001
	EDEQ total	*Parametric terms*	*Estimate*	*SE*	*t statistic*	*p-value*
		EDEQ x film category (positive)	−0.04	0.09	−0.40	0.688
		EDEQ x film category (neutral)	−0.004	0.09	−0.05	0.962
		EDEQ x film category (negative)	0.02	0.09	0.25	0.804
		*Smooth terms*	*EDF*	*RDF*	*F statistic*	*p-value*
		Time: positive films	12.31	13.51	9.03	< 0.001
		Time: neutral films	12.45	13.16	22.23	< 0.001
		Time: negative films	11.91	13.23	12.83	< 0.001
	HADS anxiety	*Parametric terms*	*Estimate*	*SE*	*t statistic*	*p-value*
		HADS anxiety x film category (positive)	−0.01	0.03	−0.29	0.771
		HADS anxiety x film category (neutral)	0.004	0.03	0.13	0.895
		HADS anxiety x film category (negative)	0.007	0.03	0.23	0.820
		*Smooth terms*	*EDF*	*RDF*	*F statistic*	*p-value*
		Time: positive films	12.31	13.50	9.03	< 0.001
		Time: neutral films	12.64	13.27	23.14	< 0.001
		Time: negative films	11.90	13.22	12.74	< 0.001
	HADS depression	*Parametric terms*	*Estimate*	*SE*	*t statistic*	*p-value*
		HADS depression x film category (positive)	−0.07	0.03	−2.31	0.021
		HADS depression x film category (neutral)	−0.05	0.03	−1.85	0.064
		HADS depression x film category (negative)	−0.04	0.03	−1.52	0.129
		*Smooth terms*	*EDF*	*RDF*	*F statistic*	*p-value*
		Time: positive films	12.27	13.48	8.98	< 0.001
		Time: neutral films	12.50	13.19	22.53	< 0.001
		Time: negative films	11.96	13.26	13.22	< 0.001
Mood ratings (Facial affect task)	BMI	*Fixed terms*	*Estimate*	*SE*	*z statistic*	*p-value*
		BMI x film category (positive)	0.004	0.03	0.13	0.894
		BMI x film category (neutral)	0.02	0.03	0.84	0.398
		BMI x film category (negative)	0.01	0.03	0.20	0.842
		Film category (neutral)	0.68	0.57	1.20	0.231
		Film category (positive)	1.58	0.57	2.77	0.006
	EDEQ total	*Fixed terms*	*Estimate*	*SE*	*z statistic*	*p-value*
		EDEQ x film category (positive)	−0.01	0.05	−0.14	0.891
		EDEQ x film category (neutral)	−0.06	0.05	−1.37	0.172
		EDEQ x film category (negative)	−0.11	0.05	−2.39	0.017
		Film category (neutral)	0.84	0.21	4.04	< 0.001
		Film category (positive)	1.18	0.22	5.46	< 0.001
	HADS anxiety	*Fixed terms*	*Estimate*	*SE*	*z statistic*	*p-value*
		HADS anxiety x film category (positive)	−0.001	0.02	−0.04	0.969
		HADS anxiety x film category (neutral)	−0.03	0.02	−1.71	0.088
		HADS anxiety x film category (negative)	−0.03	0.02	−1.73	0.084
		Film category (neutral)	1.01	0.26	3.96	< 0.001
		Film category (positive)	1.20	0.27	4.50	< 0.001
	HADS depression	*Fixed terms*	*Estimate*	*SE*	*z statistic*	*p-value*
		HADS depression x film category (positive)	−0.006	0.02	−0.37	0.709
		HADS depression x film category (neutral)	−0.02	0.02	−1.15	0.252
		HADS depression x film category (negative)	−0.02	0.02	−1.23	0.220
		Film category (neutral)	1.00	0.18	5.49	< 0.001
		Film category (positive)	1.43	0.20	7.17	< 0.001
Mood ratings (fMRI task)	BMI	*Fixed terms*	*Estimate*	*SE*	*z statistic*	*p-value*
		BMI x film category (positive)	0.02	0.03	0.87	0.384
		BMI x film category (neutral)	0.02	0.03	0.79	0.432
		BMI x film category (negative)	0.02	0.03	0.68	0.498
		Film category (neutral)	0.93	0.58	1.61	0.108
		Film category (positive)	1.60	0.58	2.77	0.006
	EDEQ total	*Fixed terms*	*Estimate*	*SE*	*z statistic*	*p-value*
		EDEQ x film category (positive)	−0.05	0.04	−1.05	0.294
		EDEQ x film category (neutral)	−0.09	0.04	−2.17	0.030
		EDEQ x film category (negative)	−0.16	0.04	−3.64	< 0.001
		Film category (neutral)	0.78	0.22	3.51	< 0.001
		Film category (positive)	1.30	0.22	5.88	< 0.001
	HADS anxiety	*Fixed terms*	*Estimate*	*SE*	*z statistic*	*p-value*
		HADS anxiety x film category (positive)	−0.04	0.02	−2.49	0.013
		HADS anxiety x film category (neutral)	−0.02	0.02	−0.98	0.325
		HADS anxiety x film category (negative)	−0.03	0.02	−1.67	0.095
		Film category (neutral)	0.88	0.26	3.38	< 0.001
		Film category (positive)	1.86	0.26	7.16	< 0.001
	HADS depression	*Fixed terms*	*Estimate*	*SE*	*z statistic*	*p*−*value*
		HADS depression x film category (positive)	−0.05	0.01	−3.45	< 0.001
		HADS depression x film category (neutral)	−0.03	0.01	−2.17	0.030
		HADS depression x film category (negative)	−0.02	0.01	−1.76	0.079
		Film category (neutral)	1.06	0.18	5.81	< 0.001
		Film category (positive)	1.91	0.18	10.51	< 0.001

#### Mood ratings

There were no significant effects of any clinical variables on the mood ratings during the Facial affect task (Table 4). There was a significant negative association between HADS depression scores and mood ratings in response to the positive films during the fMRI task (Table 4). There was also a significant negative association between EDEQ total scores and mood ratings in response to the negative films during the fMRI task.

#### BOLD response

There were no significant effects of any clinical variables on the BOLD response during the fMRI evoked emotions task.

## Discussion

This study aimed to replicate previous work showing reduced facial expressivity in underweight and weight-restored people with AN and to identify the neural mechanisms associated with these difficulties in reception of emotional communication. In addition to dampened facial expressivity, we hypothesised that the AN participants would show reduced BOLD response in the posterior, striatal, and frontal regions during the emotional films. Our hypotheses were partly confirmed. We found a non-linear effect of reduced positive facial affect and lower mood in response to the positive film stimuli in the AN group. However, we did not find significant group differences in the BOLD signal or functional connectivity during the fMRI task.

The results of the Facial affect task are in line with findings from a meta-analysis showing that people with AN display less positive facial affect in response to positive films than HC participants [5]. Although we did not replicate group differences in the expression of negative facial affect during the negative films [5], a previous mega-analysis also documented only reduced positive facial expressivity among those with AN [6]. Moreover, along the lines of our findings, previous studies have reported that people with AN not only show reduced positive facial expressivity but also lower self-reported positive affect or higher negative affect in response to positive stimuli [8, 46, 47]. Taken together, the present study strengthens previous findings by replicating them with different emotionally provoking stimuli and highlights the alteration in both subjective experience and outward expression of positive emotions in AN.

Although the fMRI task showed activation of and connectivity between regions typically associated with perception of emotional communication and subjective experience of emotions [15, 48, 49], we did not replicate previously reported alterations in BOLD response to emotional stimuli in AN [12]. Although this may raise questions about the neural underpinnings of the reduced facial expressivity in the Facial affect task, there are several possible reasons for this finding. This may be related to the fixed order of the tasks. As the facial affect task was conducted first, it may have reflected high inhibition in the AN group who may have warmed up to the task by the time of the fMRI examination. Indeed, people with AN report higher levels of behavioural and social inhibition, which are suggested to be related to difficulties in social interactions [50–52]. It is also possible that the facial affect finding is an artefact and that people with AN do not have general difficulties in reacting to emotional communication. This is supported by a recent study that examined reactions to real-life conversations of positive and negative topics [53]. The authors found no differences between the AN and HC groups in facial affect during the conversations, but those with AN were more likely to look away and not lean in towards the researcher [53]. Additionally, recent fMRI studies have increasingly reported no significant group differences between people with AN or those with lived experience of AN and HC participants in whole-brain responses to emotional faces and social interactions [54–57]. Further investigation of the role of social inhibition on reception of emotional communication is needed to shed light on this discrepancy.

The exploratory analyses revealed negative associations between depression and ED symptomatology and mood ratings during the fMRI task but not with the other outcomes. This could suggest that reception of the emotional content of the films was not generally associated with illness severity. Although some previous studies have reported that facial reactions evoked by emotional films are associated with psychopathology in AN [7, 8, 46], these findings are in line with other studies that failed to find such an association [58, 59]. These findings could also be interpreted to indicate that social-emotional difficulties in AN may not lie in reactivity to and reception of emotional communication but may be more related to interpretation. Rating ones mood requires awareness of subjective experience and internal emotional states, suggesting that ratings themselves could be part of top-down decision making and interpretation [60]. Previous studies have documented that people with AN have a greater tendency to interpret benign information as negative and are less likely to choose positive interpretations [61–63]. Still, it is worth noting that the mood ratings during the Facial affect task were not associated with any clinical variables. Therefore, further investigation of the nature of difficulties in reception of emotional communication is of interest before clinical implications can be drawn.

### Limitations

The main limitation of this study was the fact that participants with AN were included as long as they did not consider themselves to be recovered, regardless of BMI status. This resulted in a heterogeneous sample of underweight and weight-restored people with AN, which makes it difficult to determine whether the present findings generalise to the acute stage of illness instead of reflecting at least a partially weight-restored stage. Along the same lines, we did not assess autistic traits, AN subtypes, or the duration of illness. This may have impacted the results, as autism is characterised by social difficulties and long duration of AN can alter social functioning as the impact of malnutrition, isolation, and chronic stress accumulate [64]. Further investigation of the impact of autistic features, illness subtypes, and duration of illness on social-emotional processing in AN is of interest.

Another limitation of the study is that continuous mood ratings were not employed in either task. This makes it difficult to investigate associations between subjective experiences and facial expressions or brain responses. However, implementing continuous mood ratings is not without challenges. Continuous rating during fMRI affects neural responses and it can distract participants from the stimuli [13]. Collecting continuous ratings afterwards has been proposed as an alternative but the ratings may be affected by second viewing of the same stimuli [13]. Further investigation into the optimal method of acquiring continuous mood ratings is needed.

Another limitation of the present study was the sample size. Although the present findings show activation in regions typically associated with reactions to emotional films, future studies using a similar fMRI paradigm with relatively few trials would benefit from a larger sample size [65]. Additionally, including only right-handed female participants limits the generalisability of the findings. Although this was done in an attempt to reduce heterogeneity in the present study, it is worth noting that more recent evidence suggests that the exclusion of left-handed participants may not be necessary in most fMRI studies [66]. Further, EDs do not exclusively affect women [67, 68], and future studies may benefit from recruiting larger samples of all genders to explore the impact of gender.

## Conclusions

This study aimed to replicate previously reported reduced facial expressivity in women with AN and further investigate the neural underpinnings of such blunted emotional reactions using two evoked emotions tasks in and outside the MRI environment. There was a non-linear difference between the AN and HC participants in positive facial expressions in response to positive emotional films. The AN participants also reported lower mood in response to these films. No group differences were observed in the BOLD response to emotional films or functional connectivity during the fMRI task. Furthermore, only self-reported mood during the fMRI task was negatively associated with ED symptomatology and depression. There were no other significant associations with the clinical variables. Thus, the neural mechanisms that underlie the observed reduced facial expressivity and its relationship to psychopathology in AN remain unclear.

## Supplementary information

Supplementary materials
