# Supplementary material for: Evoked emotions in anorexia nervosa: neural and behavioural correlates of social-emotional processing
Source: Transl Psychiatry. 2026 Feb 19;16:128. doi: 10.1038/s41398-026-03819-8 (PMC12966300; doi:10.1038/s41398-026-03819-8)
Supplement: Supplementary file 1 — Supplementary materials [file 41398_2026_3819_MOESM1_ESM.docx]

# Film clips used in the evoked emotions tasks

Supplementary Table 1. Film clips used

| Task | Film clip | Name of original short film | Minutes | Description |
| --- | --- | --- | --- | --- |
| Behavioural evoked emotions | Film 3 | Lifeboat | 5:32 – 8:33 | Counsellor is leading an uncomfortable "tough love" -style group session. |
|  | Film 5 | Pride and Pack –Pride of Lions | 17:58 – 20:09 | Group of women are getting ready for a party when an argument about racial identity breaks out. |
|  | Film 9 | Dreaming whilst Black | 1:16 – 3:32 | People working at a set are preparing to start filming. |
|  | Film 10 | Ohio | 5:36 – 7:47 | A woman meets a man and they walk around Los Angeles. |
|  | Film 14 | Reality 2.0: Catcalling | 0:07 – 2:50 | A humorous film depicting a woman confronting a catcaller. |
|  | Film 15 | Chinese Hi-Five | 0:11 – 2:17 | Two people meet on the street and make jokes while getting to know each other. |
| fMRI evoked emotions | Film 4 | Presentation | 4:47 – 7:10 | A family dinner scene where the youngest child is taunting the older sibling. Parents come to the younger siblings defence. |
|  | Film 6 | Work | 0:18 – 2:22 | A woman at a dance class is ridiculed by the teacher for not learning the routine. |
|  | Film 11 | Reception | 0:07 – 2:24 | Two people meet at a wedding and shat about how they know the couple. |
|  | Film 12 | RPG | 0:00 – 2:10 | A group of friends are playing Dungeons and Dragons. |
|  | Film 16 | Hot mess | 0:24 – 2:49 | A humorous film depicting woman cheering up her friend after a party. |
|  | Film 18 | Standby | 0:21 – 2:26 | Two police officers start working together and become good friends. |

# fMRIprep boilerplate

Results included in this manuscript come from preprocessing performed using fMRIPrep 23.1.3 (Esteban et al. (2019); Esteban et al. (2018); RRID:SCR_016216), which is based on Nipype 1.8.6 (K. Gorgolewski et al. (2011); K. J. Gorgolewski et al. (2018); RRID:SCR_002502).

## Anatomical data preprocessing

A total of 1 T1-weighted (T1w) images were found within the input BIDS dataset.The T1-weighted (T1w) image was corrected for intensity non-uniformity (INU) with N4BiasFieldCorrection (Tustison et al. 2010), distributed with ANTs (version unknown) (Avants et al. 2008, RRID:SCR_004757), and used as T1w-reference throughout the workflow. The T1w-reference was then skull-stripped with a Nipype implementation of the antsBrainExtraction.sh workflow (from ANTs), using OASIS30ANTs as target template. Brain tissue segmentation of cerebrospinal fluid (CSF), white-matter (WM) and gray-matter (GM) was performed on the brain-extracted T1w using fast (FSL (version unknown), RRID:SCR_002823, Zhang, Brady, and Smith 2001). Brain surfaces were reconstructed using recon-all (FreeSurfer 7.3.2, RRID:SCR_001847, Dale, Fischl, and Sereno 1999), and the brain mask estimated previously was refined with a custom variation of the method to reconcile ANTs-derived and FreeSurfer-derived segmentations of the cortical gray-matter of Mindboggle (RRID:SCR_002438, Klein et al. 2017). Volume-based spatial normalization to one standard space (MNI152NLin2009cAsym) was performed through nonlinear registration with antsRegistration (ANTs (version unknown)), using brain-extracted versions of both T1w reference and the T1w template. The following template was were selected for spatial normalization and accessed with TemplateFlow (23.0.0, Ciric et al. 2022): ICBM 152 Nonlinear Asymmetrical template version 2009c [Fonov et al. (2009), RRID:SCR_008796; TemplateFlow ID: MNI152NLin2009cAsym].

## Functional data preprocessing

For each of the 2 BOLD runs found per subject (across all tasks and sessions), the following preprocessing was performed. First, a reference volume and its skull-stripped version were generated using a custom methodology of fMRIPrep. Head-motion parameters with respect to the BOLD reference (transformation matrices, and six corresponding rotation and translation parameters) are estimated before any spatiotemporal filtering using mcflirt (FSL , Jenkinson et al. 2002). BOLD runs were slice-time corrected to 0.976s (0.5 of slice acquisition range 0s-1.95s) using 3dTshift from AFNI (Cox and Hyde 1997, RRID:SCR_005927). The BOLD time-series (including slice-timing correction when applied) were resampled onto their original, native space by applying the transforms to correct for head-motion. These resampled BOLD time-series will be referred to as preprocessed BOLD in original space, or just preprocessed BOLD. The BOLD reference was then co-registered to the T1w reference using bbregister (FreeSurfer) which implements boundary-based registration (Greve and Fischl 2009). Co-registration was configured with six degrees of freedom. Several confounding time-series were calculated based on the preprocessed BOLD: framewise displacement (FD), DVARS and three region-wise global signals. FD was computed using two formulations following Power (absolute sum of relative motions, Power et al. (2014)) and Jenkinson (relative root mean square displacement between affines, Jenkinson et al. (2002)). FD and DVARS are calculated for each functional run, both using their implementations in Nipype (following the definitions by Power et al. 2014). The three global signals are extracted within the CSF, the WM, and the whole-brain masks. Additionally, a set of physiological regressors were extracted to allow for component-based noise correction (CompCor, Behzadi et al. 2007). Principal components are estimated after high-pass filtering the preprocessed BOLD time-series (using a discrete cosine filter with 128s cut-off) for the two CompCor variants: temporal (tCompCor) and anatomical (aCompCor). tCompCor components are then calculated from the top 2% variable voxels within the brain mask. For aCompCor, three probabilistic masks (CSF, WM and combined CSF+WM) are generated in anatomical space. The implementation differs from that of Behzadi et al. in that instead of eroding the masks by 2 pixels on BOLD space, a mask of pixels that likely contain a volume fraction of GM is subtracted from the aCompCor masks. This mask is obtained by dilating a GM mask extracted from the FreeSurfer’s aseg segmentation, and it ensures components are not extracted from voxels containing a minimal fraction of GM. Finally, these masks are resampled into BOLD space and binarized by thresholding at 0.99 (as in the original implementation). Components are also calculated separately within the WM and CSF masks. For each CompCor decomposition, the k components with the largest singular values are retained, such that the retained components’ time series are sufficient to explain 50 percent of variance across the nuisance mask (CSF, WM, combined, or temporal). The remaining components are dropped from consideration. The head-motion estimates calculated in the correction step were also placed within the corresponding confounds file. The confound time series derived from head motion estimates and global signals were expanded with the inclusion of temporal derivatives and quadratic terms for each (Satterthwaite et al. 2013). Frames that exceeded a threshold of 0.5 mm FD or 1.5 standardized DVARS were annotated as motion outliers. Additional nuisance timeseries are calculated by means of principal components analysis of the signal found within a thin band (crown) of voxels around the edge of the brain, as proposed by (Patriat, Reynolds, and Birn 2017). The BOLD time-series were resampled into standard space, generating a preprocessed BOLD run in MNI152NLin2009cAsym space. First, a reference volume and its skull-stripped version were generated using a custom methodology of fMRIPrep. All resamplings can be performed with a single interpolation step by composing all the pertinent transformations (i.e. head-motion transform matrices, susceptibility distortion correction when available, and co-registrations to anatomical and output spaces). Gridded (volumetric) resamplings were performed using antsApplyTransforms (ANTs), configured with Lanczos interpolation to minimize the smoothing effects of other kernels (Lanczos 1964). Non-gridded (surface) resamplings were performed using mri_vol2surf (FreeSurfer).

Many internal operations of fMRIPrep use Nilearn 0.10.1 (Abraham et al. 2014, RRID:SCR_001362), mostly within the functional processing workflow. For more details of the pipeline, see the section corresponding to workflows in fMRIPrep’s documentation.

## Copyright Waiver

The above boilerplate text was automatically generated by fMRIPrep with the express intention that users should copy and paste this text into their manuscripts unchanged. It is released under the CC0 license.

## References

Abraham, Alexandre, Fabian Pedregosa, Michael Eickenberg, Philippe Gervais, Andreas Mueller, Jean Kossaifi, Alexandre Gramfort, Bertrand Thirion, and Gael Varoquaux. 2014. “Machine Learning for Neuroimaging with Scikit-Learn.” Frontiers in Neuroinformatics 8. https://doi.org/10.3389/fninf.2014.00014.

Avants, B. B., C. L. Epstein, M. Grossman, and J. C. Gee. 2008. “Symmetric Diffeomorphic Image Registration with Cross-Correlation: Evaluating Automated Labeling of Elderly and Neurodegenerative Brain.” Medical Image Analysis 12 (1): 26–41. https://doi.org/10.1016/j.media.2007.06.004.

Behzadi, Yashar, Khaled Restom, Joy Liau, and Thomas T. Liu. 2007. “A Component Based Noise Correction Method (CompCor) for BOLD and Perfusion Based fMRI.” NeuroImage 37 (1): 90–101. https://doi.org/10.1016/j.neuroimage.2007.04.042.

Ciric, R., William H. Thompson, R. Lorenz, M. Goncalves, E. MacNicol, C. J. Markiewicz, Y. O. Halchenko, et al. 2022. “TemplateFlow: FAIR-Sharing of Multi-Scale, Multi-Species Brain Models.” Nature Methods 19: 1568–71. https://doi.org/10.1038/s41592-022-01681-2.

Cox, Robert W., and James S. Hyde. 1997. “Software Tools for Analysis and Visualization of fMRI Data.” NMR in Biomedicine 10 (4-5): 171–78. https://doi.org/10.1002/(SICI)1099-1492(199706/08)10:4/5<171::AID-NBM453>3.0.CO;2-L.

Dale, Anders M., Bruce Fischl, and Martin I. Sereno. 1999. “Cortical Surface-Based Analysis: I. Segmentation and Surface Reconstruction.” NeuroImage 9 (2): 179–94. https://doi.org/10.1006/nimg.1998.0395.

Esteban, Oscar, Ross Blair, Christopher J. Markiewicz, Shoshana L. Berleant, Craig Moodie, Feilong Ma, Ayse Ilkay Isik, et al. 2018. “fMRIPrep 23.1.3.” Software. https://doi.org/10.5281/zenodo.852659.

Esteban, Oscar, Christopher Markiewicz, Ross W Blair, Craig Moodie, Ayse Ilkay Isik, Asier Erramuzpe Aliaga, James Kent, et al. 2019. “fMRIPrep: A Robust Preprocessing Pipeline for Functional MRI.” Nature Methods 16: 111–16. https://doi.org/10.1038/s41592-018-0235-4.

Fonov, VS, AC Evans, RC McKinstry, CR Almli, and DL Collins. 2009. “Unbiased Nonlinear Average Age-Appropriate Brain Templates from Birth to Adulthood.” NeuroImage 47, Supplement 1: S102. https://doi.org/10.1016/S1053-8119(09)70884-5.

Gorgolewski, K., C. D. Burns, C. Madison, D. Clark, Y. O. Halchenko, M. L. Waskom, and S. Ghosh. 2011. “Nipype: A Flexible, Lightweight and Extensible Neuroimaging Data Processing Framework in Python.” Frontiers in Neuroinformatics 5: 13. https://doi.org/10.3389/fninf.2011.00013.

Gorgolewski, Krzysztof J., Oscar Esteban, Christopher J. Markiewicz, Erik Ziegler, David Gage Ellis, Michael Philipp Notter, Dorota Jarecka, et al. 2018. “Nipype.” Software. https://doi.org/10.5281/zenodo.596855.

Greve, Douglas N, and Bruce Fischl. 2009. “Accurate and Robust Brain Image Alignment Using Boundary-Based Registration.” NeuroImage 48 (1): 63–72. https://doi.org/10.1016/j.neuroimage.2009.06.060.

Jenkinson, Mark, Peter Bannister, Michael Brady, and Stephen Smith. 2002. “Improved Optimization for the Robust and Accurate Linear Registration and Motion Correction of Brain Images.” NeuroImage 17 (2): 825–41. https://doi.org/10.1006/nimg.2002.1132.

Klein, Arno, Satrajit S. Ghosh, Forrest S. Bao, Joachim Giard, Yrjö Häme, Eliezer Stavsky, Noah Lee, et al. 2017. “Mindboggling Morphometry of Human Brains.” PLOS Computational Biology 13 (2): e1005350. https://doi.org/10.1371/journal.pcbi.1005350.

Lanczos, C. 1964. “Evaluation of Noisy Data.” Journal of the Society for Industrial and Applied Mathematics Series B Numerical Analysis 1 (1): 76–85. https://doi.org/10.1137/0701007.

Patriat, Rémi, Richard C. Reynolds, and Rasmus M. Birn. 2017. “An Improved Model of Motion-Related Signal Changes in fMRI.” NeuroImage 144, Part A (January): 74–82. https://doi.org/10.1016/j.neuroimage.2016.08.051.

Power, Jonathan D., Anish Mitra, Timothy O. Laumann, Abraham Z. Snyder, Bradley L. Schlaggar, and Steven E. Petersen. 2014. “Methods to Detect, Characterize, and Remove Motion Artifact in Resting State fMRI.” NeuroImage 84 (Supplement C): 320–41. https://doi.org/10.1016/j.neuroimage.2013.08.048.

Satterthwaite, Theodore D., Mark A. Elliott, Raphael T. Gerraty, Kosha Ruparel, James Loughead, Monica E. Calkins, Simon B. Eickhoff, et al. 2013. “An improved framework for confound regression and filtering for control of motion artifact in the preprocessing of resting-state functional connectivity data.” NeuroImage 64 (1): 240–56. https://doi.org/10.1016/j.neuroimage.2012.08.052.

Tustison, N. J., B. B. Avants, P. A. Cook, Y. Zheng, A. Egan, P. A. Yushkevich, and J. C. Gee. 2010. “N4ITK: Improved N3 Bias Correction.” IEEE Transactions on Medical Imaging 29 (6): 1310–20. https://doi.org/10.1109/TMI.2010.2046908.

Zhang, Y., M. Brady, and S. Smith. 2001. “Segmentation of Brain MR Images Through a Hidden Markov Random Field Model and the Expectation-Maximization Algorithm.” IEEE Transactions on Medical Imaging 20 (1): 45–57. <https://doi.org/10.1109/42.906424>.

# FMRI first-level modelling

The Haemodynamic response function (HRF) was modelled using the flexible inverse logit (IL) model. The IL model can capture varying HRF shapes, including delays and early decay of the response (Lindquist et al., 2009). This makes the IL model ideal for capturing responses to brief emotional film clips where it is difficult to know in advance when the HRF is likely to start or end. The IL parameters were calibrated using the Sequential Least Squares Programming optimiser in *scipy's* *minimize* (Lawson & Hanson, 1995; Virtanen et al., 2020). The cost was calculated using Gaussian maximum likelihood estimation.

## References

Lawson, C. L., & Hanson, R. J. (1995). *Solving least squares problems*. SIAM.

Lindquist, M. A., Loh, J. M., Atlas, L. Y., & Wager, T. D. (2009). Modeling the Hemodynamic Response Function in fMRI: Efficiency, Bias and Mis-modeling. *Neuroimage*, *45*(1 Suppl), S187–S198. https://doi.org/10.1016/j.neuroimage.2008.10.065

Virtanen, P., Gommers, R., Oliphant, T. E., Haberland, M., Reddy, T., Cournapeau, D., Burovski, E., Peterson, P., Weckesser, W., Bright, J., van der Walt, S. J., Brett, M., Wilson, J., Millman, K. J., Mayorov, N., Nelson, A. R. J., Jones, E., Kern, R., Larson, E., … van Mulbregt, P. (2020). SciPy 1.0: Fundamental algorithms for scientific computing in Python. *Nature Methods*, *17*(3), 261–272. https://doi.org/10.1038/s41592-019-0686-2

Winkler, A. M., Ridgway, G. R., Webster, M. A., Smith, S. M., & Nichols, T. E. (2014). Permutation inference for the general linear model. *NeuroImage*, *92*, 381–397. https://doi.org/10.1016/j.neuroimage.2014.01.060

# Multi-subject dictionary learning atlas

Supplementary Table 2. Multi-subject dictionary learning (MSDL) atlas labels and coordinates

| MSDL atlas labels | MNI Co-ordinates | | |
| --- | --- | --- | --- |
|  | X | Y | Z |
| Left auditory | -53.28 | -8.88 | 32.36 |
| Right auditory | 53.47 | -6.49 | 27.52 |
| Striate | 1.18 | -74.54 | 10.79 |
| Left default mode network | -45.8 | -64.78 | 31.84 |
| Medial default mode network | -0.2 | -55.21 | 29.87 |
| Front default mode network | -0.15 | 51.42 | 7.58 |
| Right default mode network | 51.66 | -59.34 | 28.88 |
| Occipital posterior | 0.41 | -91.05 | 1.58 |
| Motor | -1.48 | -27.93 | 61.5 |
| Right dorsolateral prefrontal cortex | 40.1 | 20.96 | 44.72 |
| Right front pole | 37.83 | 55.49 | 1.22 |
| Right parietal | 47.53 | -52.42 | 43.06 |
| Right posterior temporal | 62.53 | -32.99 | -9.14 |
| Basal ganglia | -0.91 | -2.75 | 6.15 |
| Left parietal | -41.66 | -59.04 | 44.61 |
| Left dorsolateral prefrontal cortex | -39.04 | 19.28 | 43.27 |
| Left frontal pole | -40.08 | 50.65 | 0.81 |
| Left intraparietal sulcus | -29.39 | -59.43 | 44.2 |
| Right intraparietal sulcus | 31.6 | -58.09 | 45.69 |
| Left lateral occipital complex | -30.54 | -85.14 | 9.1 |
| Visual | -24.29 | -74.28 | -11.74 |
| Right lateral occipital complex | 33.4 | -77.96 | 4.31 |
| Dorsal anterior cingulate cortex | -28.17 | 46.32 | 21.56 |
| Ventral anterior cingulate cortex | -0.45 | 34.06 | 20.73 |
| Right anterior insula | 28.38 | 47.72 | 22.13 |
| Left superior temporal sulcus | -52.12 | -17.92 | 13.28 |
| Right superior temporal sulcus | 52.61 | -13.65 | 12.11 |
| Left temporoparietal junction | -55.52 | -43.77 | 10.08 |
| Broca | -48.66 | 25.11 | 5.7 |
| Superior frontal sulcus | -3.39 | 17.19 | 63.52 |
| Right temporoparietal junction | 54.42 | -29.5 | -2.72 |
| Right pars opercularis | 52.38 | 29.39 | 2.93 |
| Cerebellum | 1.05 | -58.49 | -23.91 |
| Dorsal posterior cingulate cortex | -1.44 | -59.12 | 55.25 |
| Left insula | -41.33 | 13.63 | 2.7 |
| Cingulate | 1.05 | 9.2 | 46.43 |
| Right insula | 43.01 | 14.3 | 2.79 |
| Left anterior intraparietal sulcus | -47.85 | -32.07 | 41.9 |
| Right anterior intraparietal sulcus | 48.36 | -29.04 | 43.13 |

# Association between mood ratings and BOLD signal

## Statistical analysis

We conducted exploratory mixed effects analyses to examine associations between subjective mood ratings and BOLD response and group by mood rating interaction. The analyses we conducted using Permutation Analysis of Linear Models (PALM) in FSL (Winkler et al., 2014). The mood ratings were centred prior to analysis.

## Results

All clusters where the BOLD signal was significantly associated with the mood ratings were within the regions that were associated with film categories above (Supplementary table 3, Supplementary figure 1). Multiple clusters spanning the posterior cingulate bilaterally, right superior temporal cortex, and right insula had a positive association with the mood ratings. Only one cluster in the left precuneus had a negative association with the mood ratings. There were no significant differences between the groups in the association between mood ratings and BOLD responses to the films.

Supplementary Table 3. Association between mood ratings and BOLD signal

| Cluster | Peak coordinates | | | Cluster size (mm^3^) | Test statistics | | Regions (AAL) |
| --- | --- | --- | --- | --- | --- | --- | --- |
|  | X | Y | Z |  | Peak | Cluster mean |  |
| 1 | -6.5 | -38.8 | 33.7 | 2598 | t = 4.89, p < 0.001 | t = 3.56, p < 0.001 | R Middle Cingulate  L Middle Cingulate  L Posterior Cingulate |
| 2 | -36.5 | -31.3 | 13.9 | 278 | t = 5.38, p < 0.001 | t = 4.00, p = 0.002 | L Rolandic operculum |
| 3 | 46.0 | -23.8 | 10.6 | 696 | t = 4.48, p < 0.001 | t = 3.88, p = 0.001 | R Heschl’s gyrus  R Superior temporal cortex  R Insula |
|  | 34.8 | -27.5 | 13.9 |  |  |  |  |
|  | 42.3 | -31.3 | 13.9 |  |  |  |  |
| 4 | -6.5 | -50.0 | 13.9 | 464 | t = 4.71, p = 0.001 | t = 3.66, p = 0.003 | L Precuneus |
|  | -6.5 | -61.3 | 17.2 |  |  |  |  |
| 5 | 49.8 | -12.5 | 7.3 | 371 | t = 4.50, p = 0.001 | t = 3.70, p = 0.003 | R Heschl’s gyrus  R Superior temporal cortex  R Rolandic operculum |
| 6 | -2.8 | -57.5 | 50.2 | 278 | t = -4.41, p = 0.001 | t = -3.91, p = 0.003 | L Precuneus |
| 7 | 42.3 | -68.8 | 40.3 | 278 | t = 4.57, p = 0.002 | t = 4.00, p = 0.003 | R Angular gyrus |

Supplementary Figure 1. Association between mood ratings and BOLD response during the evoked emotions task.


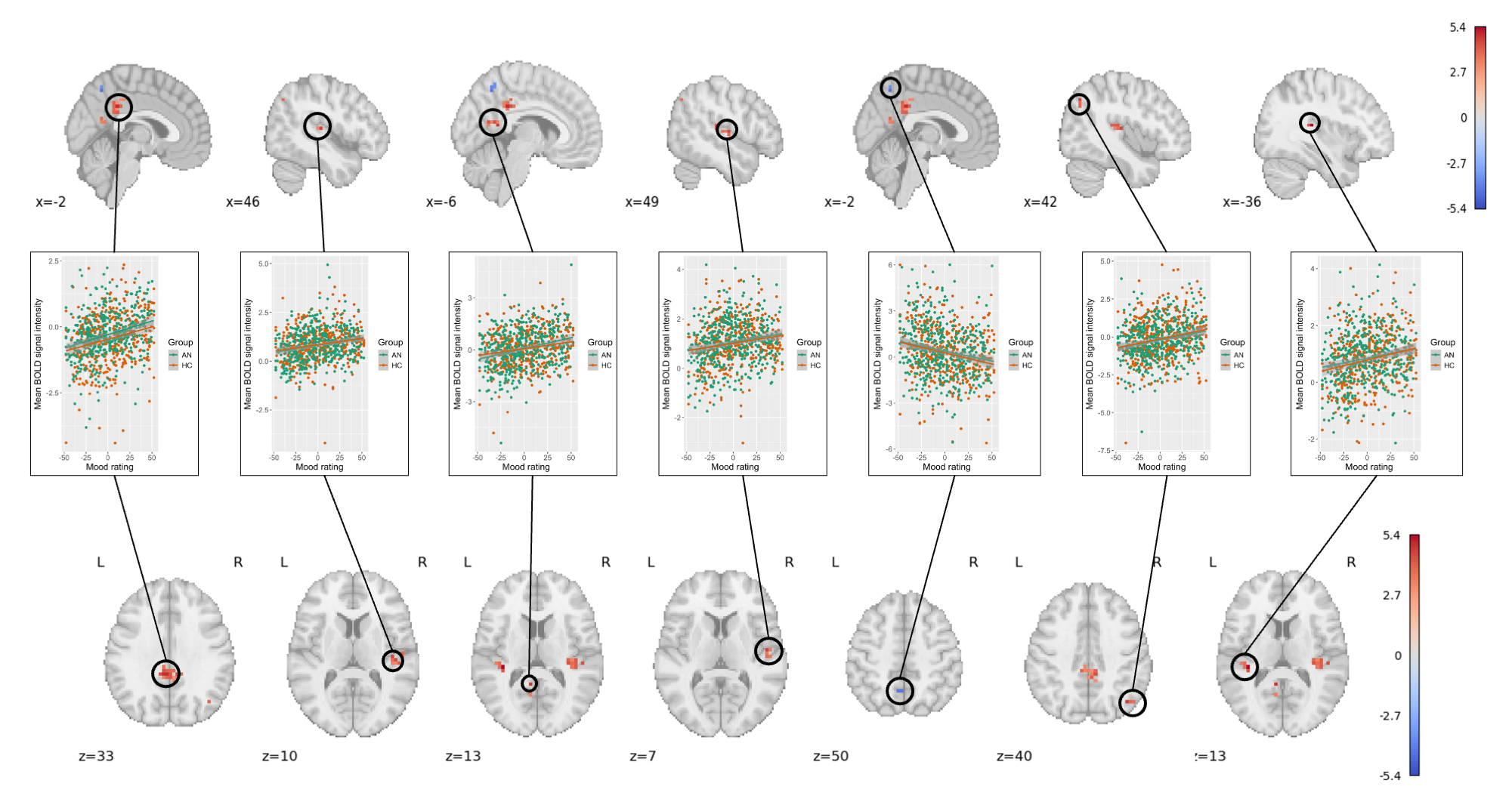


Sagittal and axial view of t-statistic clusters showing the association between subjective mood ratings and BOLD response to the film clips. The scatter plots show associations between the mood ratings and cluster mean BOLD response for HC and AN participants. The results are shown at p < 0.005 after FWER-correction.

# Model comparisons

Supplementary Table 4. Positive AUs GAMM comparisons

| Model | AIC | X^2^ test | |
| --- | --- | --- | --- |
|  |  | Reduction in residual deviance | p-value |
| Null model | -2415384 | - | - |
| No-interaction model | -2419368 | 3997.6 | < 0.001 |
| Full model | -2420150 | 830.0 | < 0.001 |

AIC = Akaike information criterion

Supplementary Table 5. Negative AUs GAMM comparisons

| Model | AIC | X^2^ test | |
| --- | --- | --- | --- |
|  |  | Reduction in residual deviance | p-value |
| Null model | -495861.7 | - | - |
| No-interaction model | -496950.1 | 1112.91 | < 0.001 |
| Full model | -497223.3 | 306.64 | < 0.001 |

AIC = Akaike information criterion

Supplementary Table 6. Facial affect task mood ratings model comparisons

| Model | AIC | X^2^ test | |
| --- | --- | --- | --- |
|  |  | X^2^ statistic | p-value |
| Null model | 2372.28 | - | - |
| No-interaction model | 1769.76 | 608.52 | < 0.001 |
| Full model | 1763.73 | 10.03 | 0.007 |

AIC = Akaike information criterion

Supplementary Table 7. fMRI task mood ratings model comparisons

| Model | AIC | X^2^ test | |
| --- | --- | --- | --- |
|  |  | X^2^ statistic | p-value |
| Null model | 2388.82 | - | - |
| No-interaction model | 1709.00 | 687.82 | < 0.001 |
| Full model | 1710.17 | 2.82 | 0.244 |

AIC = Akaike information criterion

# Facial affect and fMRI task mood ratings

There was a significant effect of film category, a group by film category interaction, and a task by film category interaction (Supplementary Table 8). The post-hoc comparisons showed that the HC group rated their mood higher than the AN group after the positive films (z=4.91, p< 0.001) and the neutral films across tasks (z=2.07, p=0.04). There was no group difference in mood ratings after negative films (z=1.35, p=0.176) across tasks. Across tasks, both groups rated their mood higher after the positive films than the neutral (AN: z=12.11, p<0.001; HC: z=15.79, p<0.001) or negative films (AN: z=29.65, p<0.001; HC: z=33.72, p<0.001). Both groups also rated their mood higher after the neutral films than after the negative films (AN: z=19.64, p<0.001; HC: z=20.57, p<0.001).

Post-hoc exploration of the task by film category interaction showed that participants rated their mood higher in response to the positive films during the fMRI task than the Facial affect task (z=2.75, p=0.006). There were no significant differences between the tasks in mood ratings in response to the neutral (z=0.64, p=0.520) or negative films (z=0.45, p=0.653). During both tasks participants rated their mood higher in response to the positive films than the neutral (Facial affect task: z=12.42, p<0.001; fMRI task: z=15.42, p<0.001) or negative films (Facial affect task: z=29.71, p<0.001; fMRI task: z=33.70, p<0.001). Participants also rated their mood higher after then neutral than negative films in both tasks (Facial affect task: z=19.98, p<0.001; fMRI task: z=20.19, p<0.001).

Supplementary Table 8. Effect of task, group and film category on mood ratings

| Fixed effect | Estimate (SE) | Test statistic | p-value |
| --- | --- | --- | --- |
| Group | 0.07 (0.08) | z = 0.83 | p = 0.407 |
| Task | -0.04 (0.07) | z = -0.55 | p = 0.580 |
| Film category (neutral) | 1.03 (0.07) | z = 13.83 | p < 0.001 |
| Film category (positive) | 1.59 (0.08) | z = 19.85 | p < 0.001 |
| Group x Task | 0.03 (0.10) | z = 0.33 | p = 0.743 |
| Group x Film category (neutral) | 0.03 (0.10) | z = 0.66 | p = 0.510 |
| Group x Film category (positive) | 0.32 (0.11) | z = 2.89 | p = 0.004 |
| Task x Film category (neutral) | 0.01 (0.10) | z = 0.13 | p = 0.899 |
| Task x Film category (positive) | 0.25 (0.11) | z = 2.27 | p = 0.023 |
| Group x Task x Film category (neutral) | -0.05 (0.15) | z = -0.31 | p = 0.754 |
| Group x Task x Film category (positive) | -0.14 (0.16) | z = -0.87 | p = 0.387 |

SE = standard error

# fMRI evoked emotions

Supplementary Figure 1. Effect of film category: cluster mean BOLD signal intensity plots


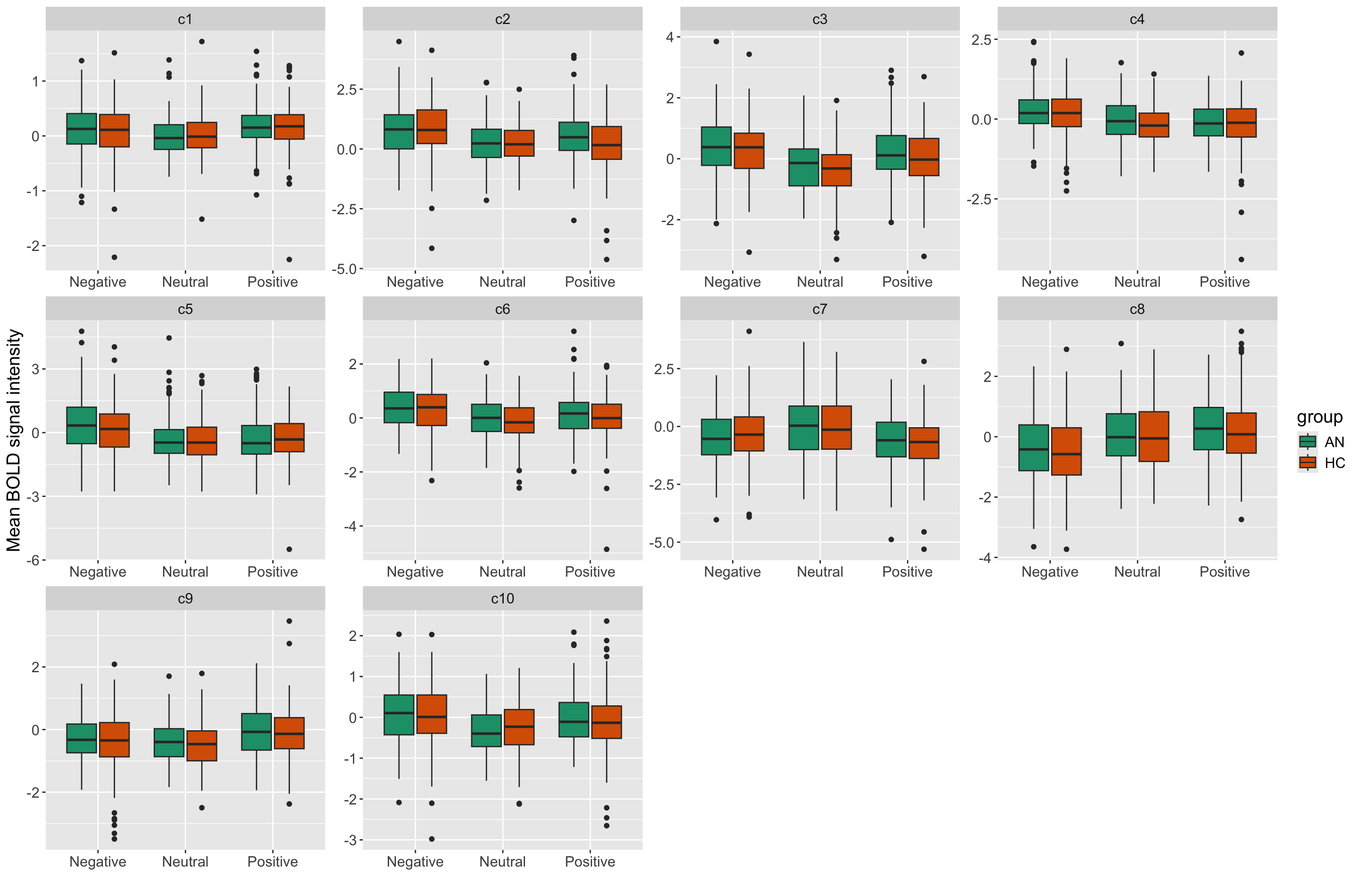


BOLD = blood oxygenation level dependent; AN = anorexia nervosa; HC = healthy comparison

Supplementary Figure 2. Negative vs neutral films: cluster mean BOLD signal intensity plots


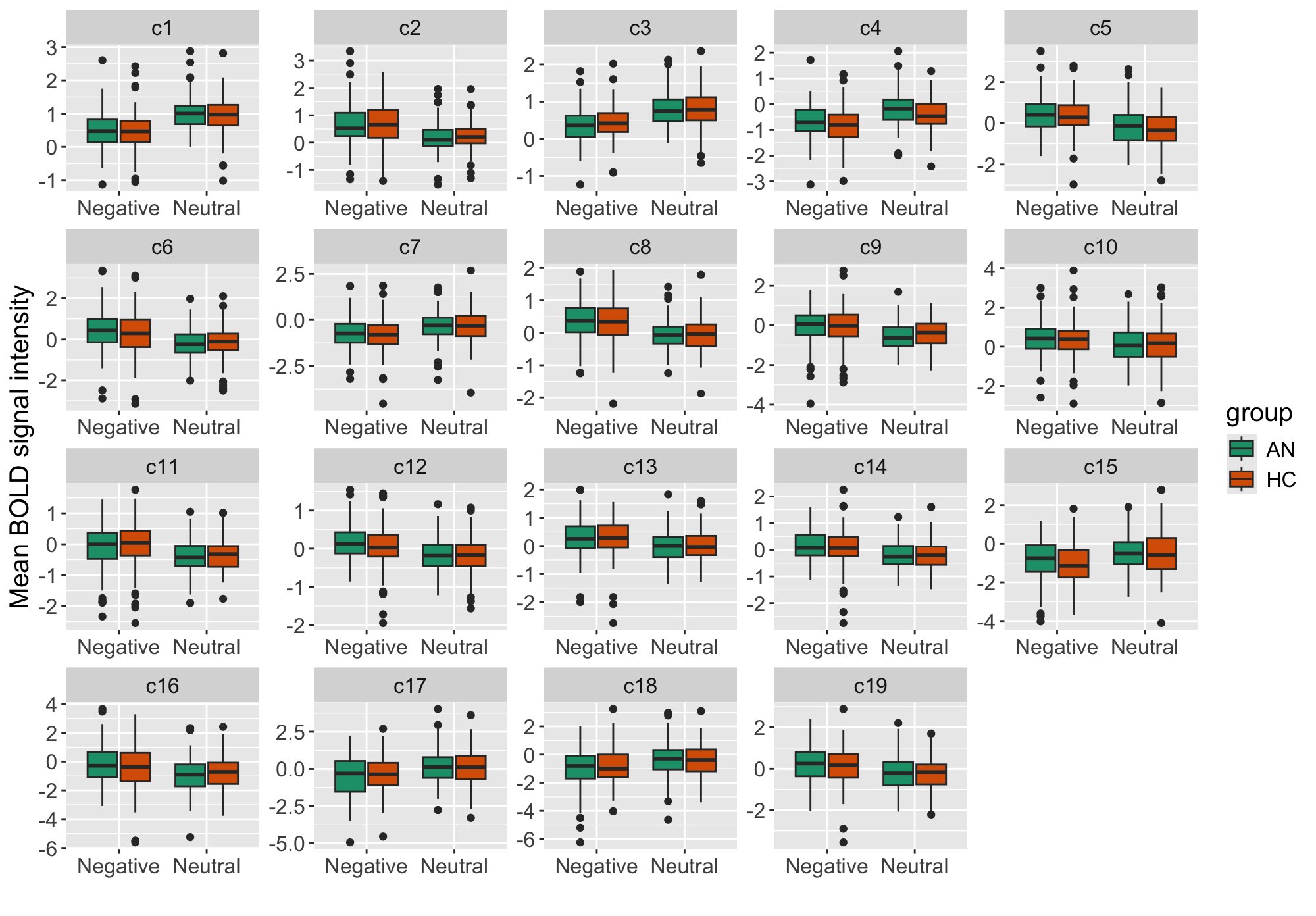


BOLD = blood oxygenation level dependent; AN = anorexia nervosa; HC = healthy comparison

Supplementary Figure 3. Positive vs Neutral: cluster mean BOLD signal intensity plots


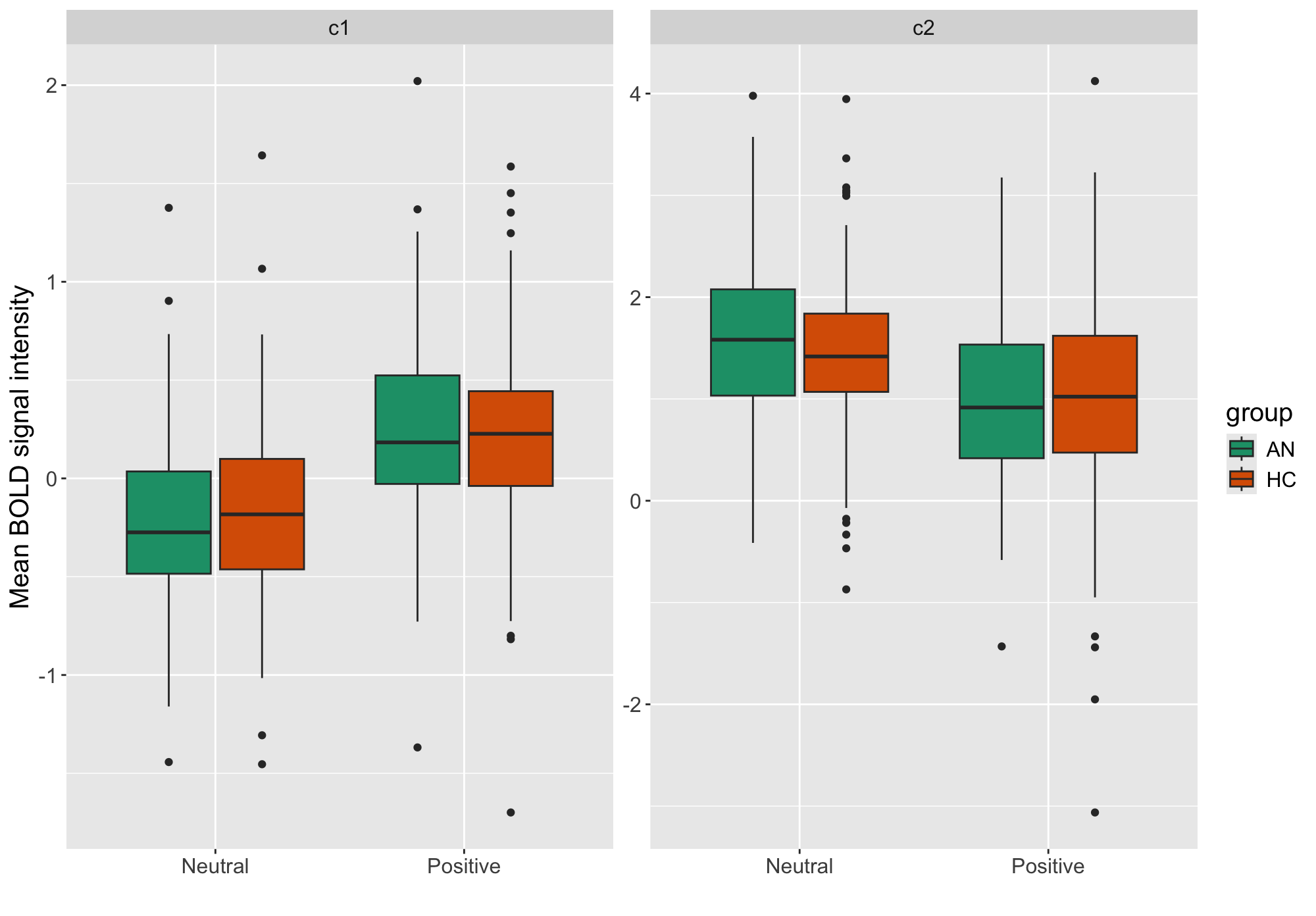


BOLD = blood oxygenation level dependent; AN = anorexia nervosa; HC = healthy comparison

Supplementary Figure 4. Negative vs Positive: cluster mean BOLD signal intensity plots


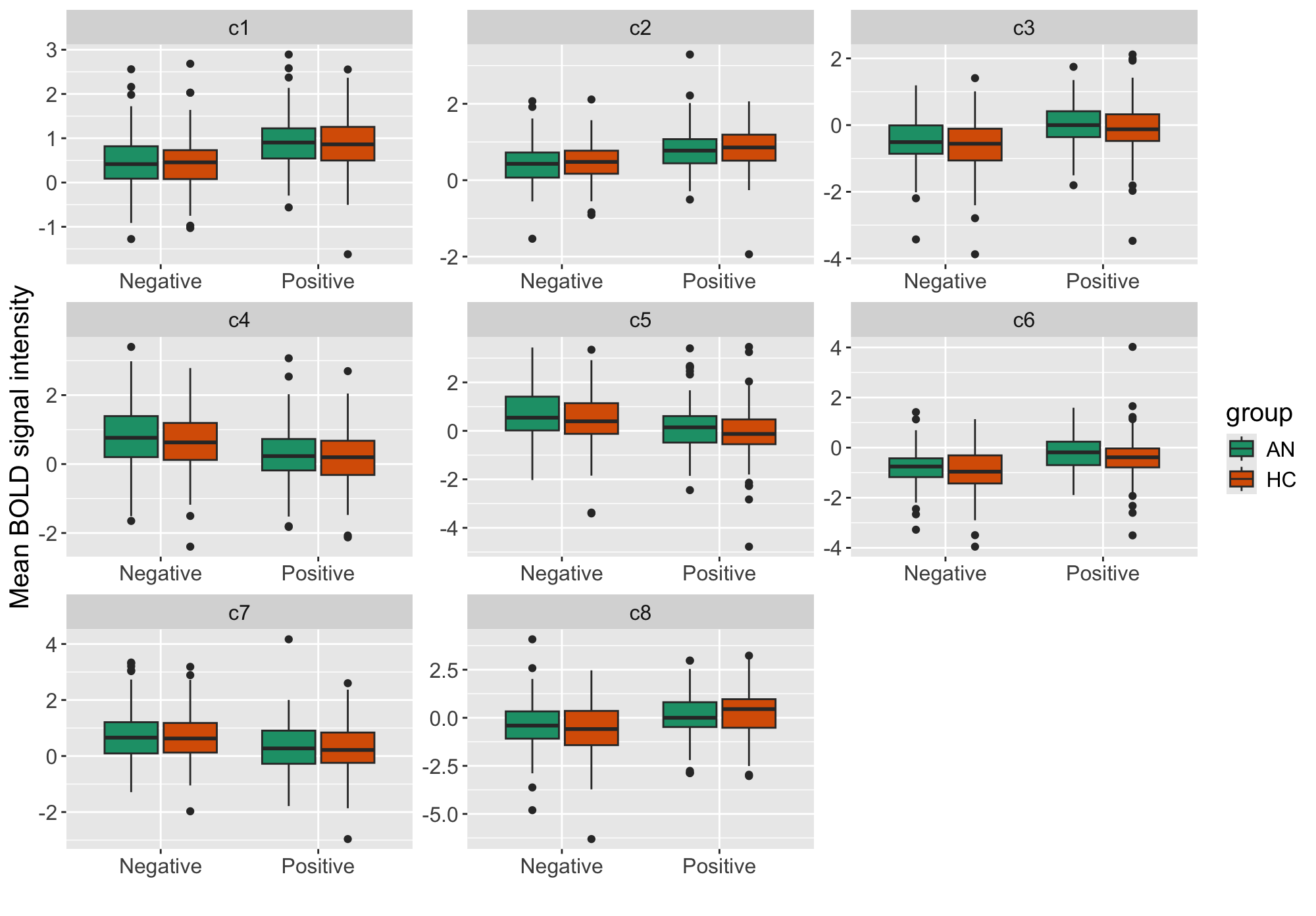


BOLD = blood oxygenation level dependent; AN = anorexia nervosa; HC = healthy comparison

# Functional connectivity during the evoked emotion task

Supplementary Table 9. Functional connectivity during the evoked emotions task

|  | From | To | Test statistic | p-value |
| --- | --- | --- | --- | --- |
| Effect of film category | Left superior temporal sulcus | Occipital posterior | F = 38.9786 | p < 0.001 |
|  | Right superior temporal sulcus | Occipital posterior | F = 34.3285 | p < 0.001 |
|  | Right anterior insula | Right intraparietal sulcus | F = 22.136 | p < 0.001 |
|  | Dorsal anterior cingulate cortex | Right intraparietal sulcus | F = 22.5455 | p < 0.001 |
|  | Right intraparietal sulcus | Left superior temporal sulcus | F = 21.9327 | p < 0.001 |
|  | Left lateral occipital complex | Occipital posterior | F = 52.5453 | p < 0.001 |
|  | Right superior temporal sulcus | Striate | F = 30.3855 | p < 0.001 |
|  | Visual | Right insula | F = 24.4196 | p < 0.001 |
|  | Visual | Left insula | F = 21.9629 | p < 0.001 |
|  | Visual | Occipital posterior | F = 20.5719 | p < 0.001 |
|  | Right superior temporal sulcus | Visual | 22.5897 | p < 0.001 |
|  | Left superior temporal sulcus | Striate | F = 21.3487 | p < 0.001 |
|  | Right intraparietal sulcus | Right temporoparietal junction | F = 17.126 | p < 0.001 |
|  | Right default mode network | Right superior temporal sulcus | F = 20.6963 | p < 0.001 |
|  | Right default mode network | Left superior temporal sulcus | F = 18.1292 | p < 0.001 |
|  | Right lateral occipital complex | Occipital posterior | F = 22.3689 | p < 0.001 |
|  | Right intraparietal sulcus | Right insula | F = 15.8704 | p < 0.001 |
|  | Right intraparietal sulcus | Right superior temporal sulcus | F = 16.6628 | p < 0.001 |
|  | Left superior temporal sulcus | Visual | F = 16.938 | p < 0.001 |
|  | Dorsal posterior cingulate cortex | Basal ganglia | F = 16.9076 | p < 0.001 |
|  | Right temporoparietal junction | Striate | F = 15.0746 | p = 0.001 |
|  | Visual | Medial default mode network | F = 14.2865 | p = 0.001 |
|  | Right lateral occipital complex | Right insula | F = 14.026 | p = 0.002 |
|  | Right intraparietal sulcus | Left temporoparietal junction | F = 13.7726 | p = 0.002 |
|  | Left temporoparietal junction | Left anterior intraparietal sulcus | F = 13.6269 | p = 0.002 |
|  | Occipital posterior | Right temporoparietal junction | F = 13.5174 | p = 0.003 |
|  | Occipital posterior | Cingulate | F = 13.4681 | p = 0.003 |
| Negative > Neutral | Left lateral occipital complex | Occipital posterior | t = -10.13 | p < 0.001 |
|  | Visual | Occipital posterior | t = -5.73 | p < 0.001 |
|  | Right superior temporal sulcus | Visual | t = 5.73 | p < 0.001 |
|  | Right lateral occipital complex | Occipital posterior | t = -6.02 | p < 0.001 |
|  | Left superior temporal sulcus | Visual | t = 5.41 | p < 0.001 |
|  | Dorsal posterior cingulate cortex | Basal ganglia | t = 5.80 | p < 0.001 |
| Positive > Neutral | Right superior temporal sulcus | Occipital posterior | t = -5.50 | p < 0.001 |
|  | Left superior temporal sulcus | Occipital posterior | t = 8.40 | p < 0.001 |
|  | Right superior temporal sulcus | Occipital posterior | t = 7.77 | p < 0.001 |
|  | Right default mode network | Right superior temporal sulcus | t = 5.84 | p < 0.001 |
|  | Right default mode network | Left superior temporal sulcus | t = 5.49 | p < 0.001 |
|  | Occipital posterior | Cingulate | t = -5.19 | p = 0.001 |
|  | Right anterior insula | Right intraparietal sulcus | t = 5.08 | p = 0.001 |
|  | Dorsal anterior cingulate cortex | Right intraparietal sulcus | t = 5.06 | p = 0.001 |
| Negative > Positive | Left superior temporal sulcus | Occipital posterior | t = -6.59 | p < 0.001 |
|  | Right superior temporal sulcus | Occipital posterior | t = -6.40 | p < 0.001 |
|  | Right anterior insula | Right intraparietal sulcus | t = -6.23 | p < 0.001 |
|  | Dorsal anterior cingulate cortex | Right intraparietal sulcus | t = -6.33 | p < 0.001 |
|  | Right intraparietal sulcus | Left superior temporal sulcus | t = -6.49 | p < 0.001 |
|  | Right intraparietal sulcus | Right insula | t = 5.36 | p < 0.001 |
|  | Left lateral occipital complex | Occipital posterior | t = -6.46 | p < 0.001 |
|  | Right intraparietal sulcus | Right superior temporal sulcus | t = -5.76 | p < 0.001 |
|  | Right superior temporal sulcus | Striate | t = 7.73 | p < 0.001 |
|  | Visual | Right insula | t = 6.96 | p < 0.001 |
|  | Visual | Left insula | t = 6.62 | p < 0.001 |
|  | Visual | Occipital posterior | t = -5.37 | p < 0.001 |
|  | Right superior temporal sulcus | Visual | t = 5.94 | p < 0.001 |
|  | Left superior temporal sulcus | Striate | t = 6.44 | p < 0.001 |
|  | Right intraparietal sulcus | Right temporoparietal junction | t = -5.83 | p < 0.001 |
|  | Right default mode network | Right superior temporal sulcus | t = -5.29 | p < 0.001 |
|  | Right lateral occipital complex | Right insula | t = 5.21 | p < 0.001 |
|  | Visual | Medial default mode network | t = -5.144 | p = 0.001 |
|  | Right intraparietal sulcus | Left temporoparietal junction | t = -5.13 | p = 0.001 |
|  | Right temporoparietal junction | Striate | t = 5.03 | p = 0.001 |
|  | Occipital posterior | Left parietal | t = -4.96 | p = 0.002 |
|  | Right default mode network | Left superior temporal sulcus | t = -4.86 | p = 0.003 |
|  | Right dorsolateral prefrontal cortex | Right superior temporal sulcus | t = -4.87 | p = 0.003 |
